# Supplementary material for: Multi-scale inference of genetic trait architecture using biologically annotated neural networks
Source: PLoS Genet. 2021 Aug 19;17(8):e1009754. doi: 10.1371/journal.pgen.1009754 (PMC8407593; doi:10.1371/journal.pgen.1009754)
Supplement: S1 Fig — (A) The BANN-SS framework requires a J-dimensional vector of SNP-level GWA marginal effect size (OLS) estimates θ^=(θ^1,…,θ^J); an empirical J × J linkage disequilibrium (LD) matrix R = [r1, …, rJ], where rj = [r(xj, x1), …, r(xj, xJ)] is a vector of correlation coefficients between the j-th SNP and all other SNPs in the study; and a list of G-predefined SNP-sets {S1,…,SG}. In this work, SNP-sets are defined as genes and intergenic regions (between genes) given by the NCBI’s Reference Sequence (RefSeq) database in the UCSC Genome Browser [50]. (B) A partially connected Bayesian neural network is constructed based on the annotated SNP groups. In the first hidden layer, only SNPs within the boundary of a gene are connected to the same node. Similarly, SNPs within the same intergenic region between genes are connected to the same node. Completing this specification for all SNPs gives the hidden layer the natural interpretation of being the “SNP-set” layer. (C) The hierarchical nature of the network is represented as nonlinear regression model. The corresponding weights in both the SNP (θ) and SNP-set (w) layers are treated as random variables with biologically motivated sparse prior distributions. Posterior inclusion probabilities PIP(j) ≡ Pr[θj ≠ 0 | y, X] and PIP(g) ≡ Pr[wg ≠ 0 | y, X, θg] summarize associations at the SNP and SNP-set level, respectively. The BANN-SS framework uses the same variational inference procedure that is used when we have access to individual-level data. (PDF) [file pgen.1009754.s001.pdf]

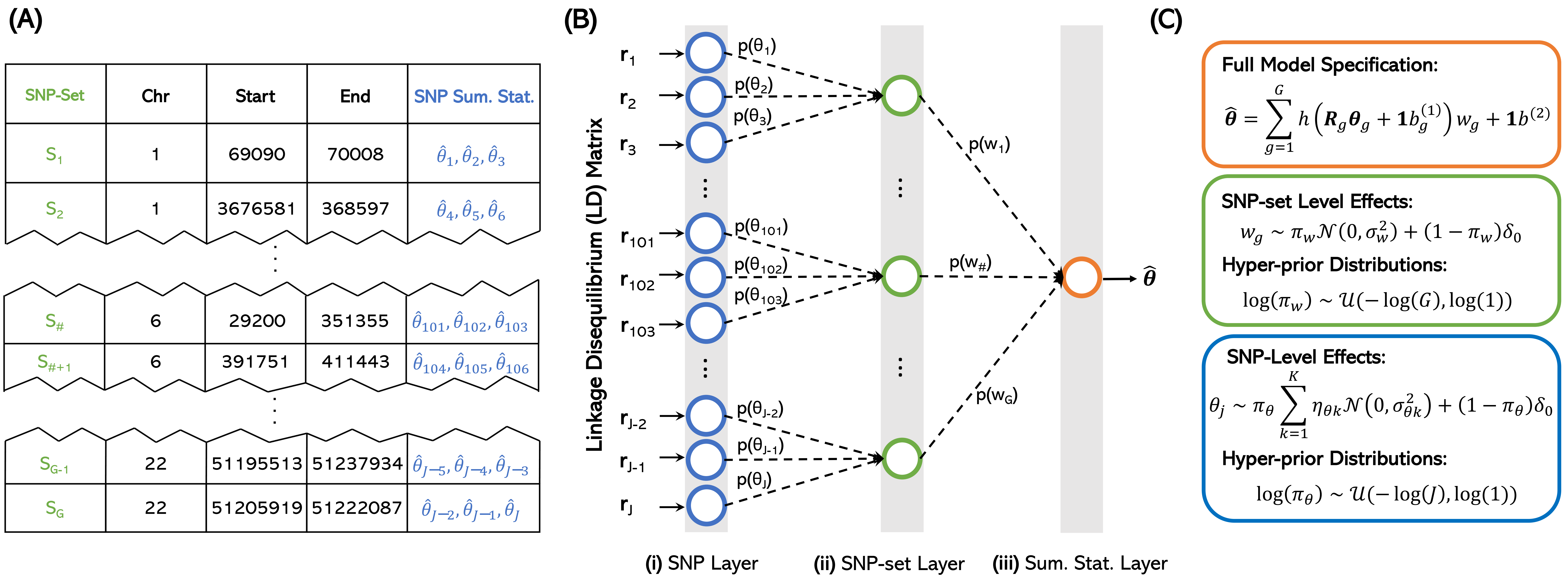

(A)
SNP-Set
Chr
Start
End
SNP Sum. Stat.
S1 1 69090 70008 $\hat{\theta}_1, \hat{\theta}_2, \hat{\theta}_3$
S2 1 3676581 368597 $\hat{\theta}_4, \hat{\theta}_5, \hat{\theta}_6$
S# 6 29200 351355 $\hat{\theta}_{101}, \hat{\theta}_{102}, \hat{\theta}_{103}$
S#+1 6 391751 411443 $\hat{\theta}_{104}, \hat{\theta}_{105}, \hat{\theta}_{106}$
SG-1 22 51195513 51237934 $\hat{\theta}_{J-5}, \hat{\theta}_{J-4}, \hat{\theta}_{J-3}$
SG 22 51205919 51222087 $\hat{\theta}_{J-2}, \hat{\theta}_{J-1}, \hat{\theta}_J$
(B)
Linkage Disequilibrium (LD) Matrix
r_1 r_2 r_3 r_{101} r_{102} r_{103} r_{J-2} r_{J-1} r_J
$p(\theta_1)$ $p(\theta_2)$ $p(\theta_3)$ $p(\theta_{101})$ $p(\theta_{102})$ $p(\theta_{103})$ $p(\theta_{J-2})$ $p(\theta_{J-1})$ $p(\theta_J)$
$p(w_1)$ $p(w_\#)$ $p(w_G)$
$\hat{\boldsymbol{\theta}}$
(i) SNP Layer
(ii) SNP-set Layer
(iii) Sum. Stat. Layer
(C)
Full Model Specification:
$\hat{\boldsymbol{\theta}} = \sum_{g=1}^{G} h\left(\boldsymbol{R}_g \boldsymbol{\theta}_g + \mathbf{1} b_g^{(1)}\right) w_g + \mathbf{1} b^{(2)}$
SNP-set Level Effects:
$w_g \sim \pi_w \mathcal{N}(0, \sigma_w^2) + (1 - \pi_w)\delta_0$
Hyper-prior Distributions:
$\log(\pi_w) \sim \mathcal{U}(-\log(G), \log(1))$
SNP-Level Effects:
$\theta_j \sim \pi_\theta \sum_{k=1}^{K} \eta_{\theta k} \mathcal{N}(0, \sigma_{\theta k}^2) + (1 - \pi_\theta)\delta_0$
Hyper-prior Distributions:
$\log(\pi_\theta) \sim \mathcal{U}(-\log(J), \log(1))$
