# Supplementary material for: Multi-scale inference of genetic trait architecture using biologically annotated neural networks
Source: PLoS Genet. 2021 Aug 19;17(8):e1009754. doi: 10.1371/journal.pgen.1009754 (PMC8407593; doi:10.1371/journal.pgen.1009754)
Supplement: S12 Fig — Here, quantitative traits are simulated to have broad-sense heritability of H2 = 0.6 with only contributions from additive effects (i.e., ρ = 1). In these simulations, traits were generated while using the top ten principal components (PCs) of the genotype matrix as covariates. We show precision versus recall for two different trait architectures: (A, B) sparse where only 1% of SNP-sets are enriched for the trait; and (C, D) polygenic where 10% of SNP-sets are enriched. We then set the number of causal SNPs with non-zero effects to be 1% and 10% of all SNPs located within the selected enriched SNP-sets, respectively. To derive results, the full genotype matrix and phenotypic vector are given to the BANNs model and all competing methods that require individual-level data. For the BANN-SS model and other competing methods that take GWA summary statistics, we compute standard GWA SNP-level effect sizes and P-values (estimated using ordinary least squares). (A, C) Competing SNP-level mapping approaches include: CAVIAR [45], SuSiE [46], and FINEMAP [44]. The software for SuSiE requires an input ℓ which fixes the maximum number of causal SNPs in the model. We display results when this input number is high (ℓ = 3000) and when this input number is low (ℓ = 10). (B, D) Competing SNP-set mapping approaches include: RSS [26], PEGASUS [25], GBJ [27], SKAT [21], GSEA [43], and MAGMA [23]. Note that, for traits with sparse architectures, the top ranked SNPs and SNP-sets are always true positives, and therefore the minimal recall is not 0. All results are based on 100 replicates (see S1 Text). (PDF) [file pgen.1009754.s012.pdf]

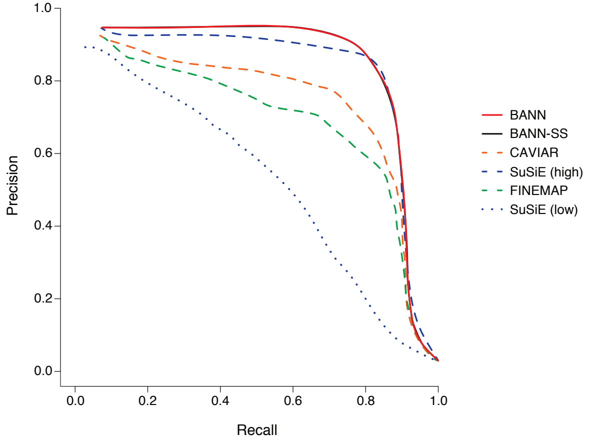

(A) SNP Methods (Sparse Traits)

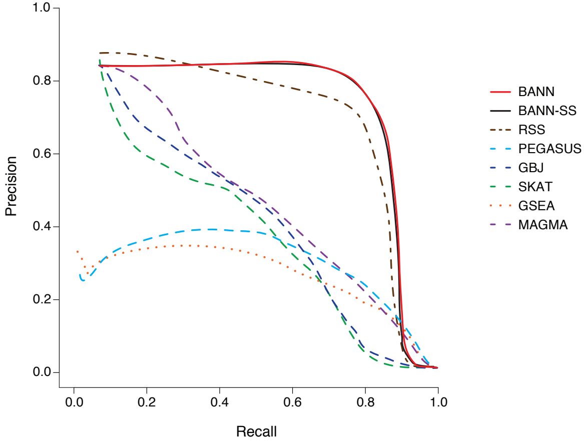

(B) SNP-Set Methods (Sparse Traits)

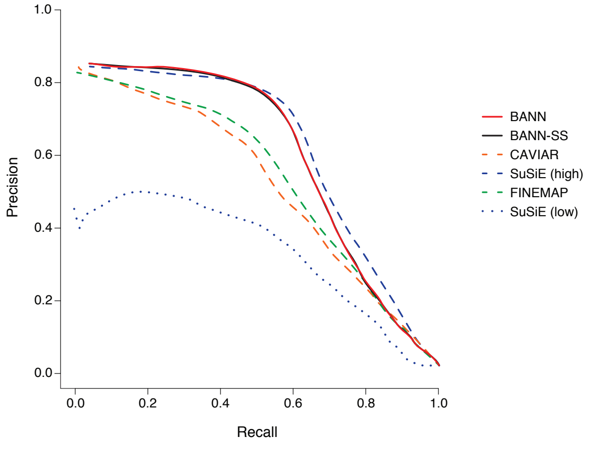

(C) SNP Methods (Polygenic Traits)

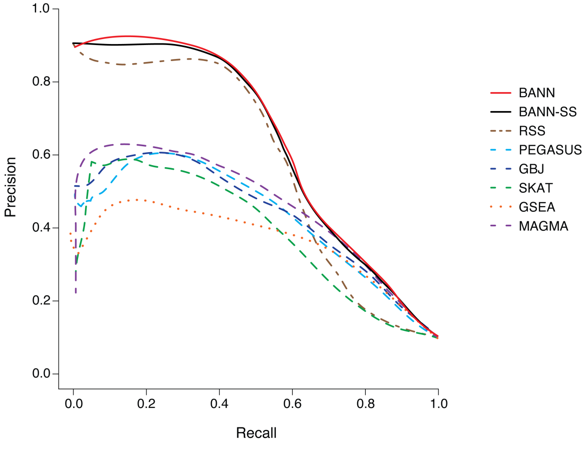

(D) SNP-Set Methods (Polygenic Traits)
