# Supplementary material for: Multi-scale inference of genetic trait architecture using biologically annotated neural networks
Source: PLoS Genet. 2021 Aug 19;17(8):e1009754. doi: 10.1371/journal.pgen.1009754 (PMC8407593; doi:10.1371/journal.pgen.1009754)
Supplement: S17 Fig — Quantitative traits are simulated to have broad-sense heritability of H2 = 0.2 with only contributions from additive effects set (i.e., ρ = 1). We consider two different trait architectures: (A, B) sparse where only 1% of SNP-sets are enriched for the trait; and (C, D) polygenic where 10% of SNP-sets are enriched. We set the number of causal SNPs with non-zero effects to be ∼ 1% and ∼ 10% of all SNPs located within the enriched SNP-sets, respectively. Results are shown comparing the posterior inclusion probabilities (PIPs) derived by the BANNs model fit with individual-level data on the x-axis and (A, C) SuSiE [46] and (B, D) RSS [26] on the y-axis, respectively. Here, SuSie is fit while assuming a high maximum number of causal SNPs (ℓ = 3000). The blue horizontal and vertical dashed lines are marked at the “median probability criterion” (i.e., PIPs for SNPs and SNP-sets greater than 0.5) [57]. True positive causal variants used to generate the synthetic phenotypes are colored in red, while non-causal variants are given in grey. SNPs and SNP-sets in the top right quadrant are selected by both approaches; while, elements in the bottom right and top left quadrants are uniquely identified by BANNs and SuSie/RSS, respectively. Each plot combines results from 100 simulated replicates (see S1 Text). (PDF) [file pgen.1009754.s017.pdf]

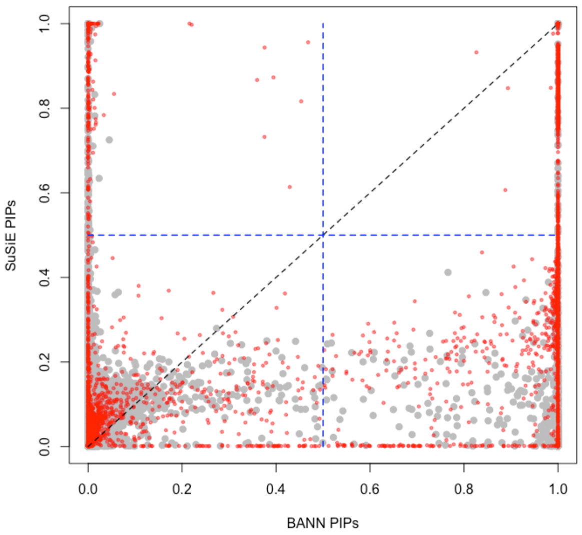

(A) SNP-Level Methods (Sparse Traits)

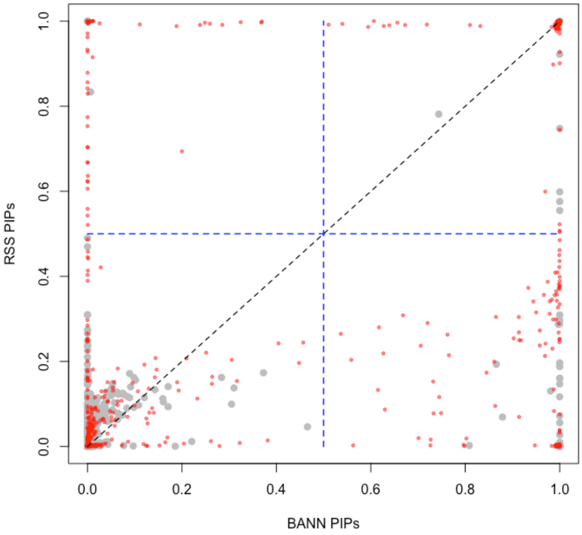

(B) SNP-Set Methods (Sparse Traits)

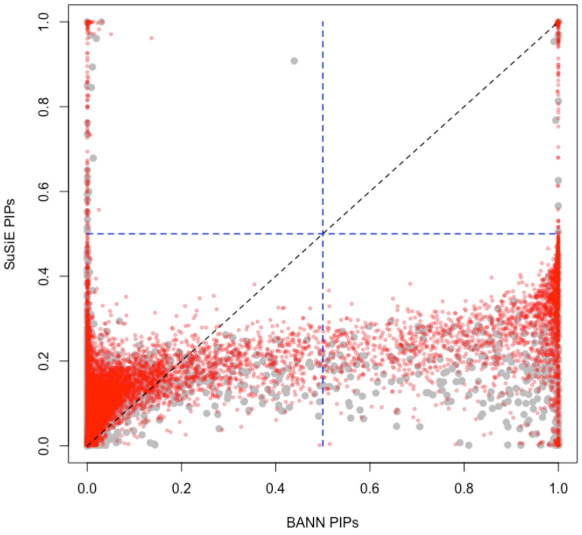

(C) SNP-Level Methods (Polygenic Traits)

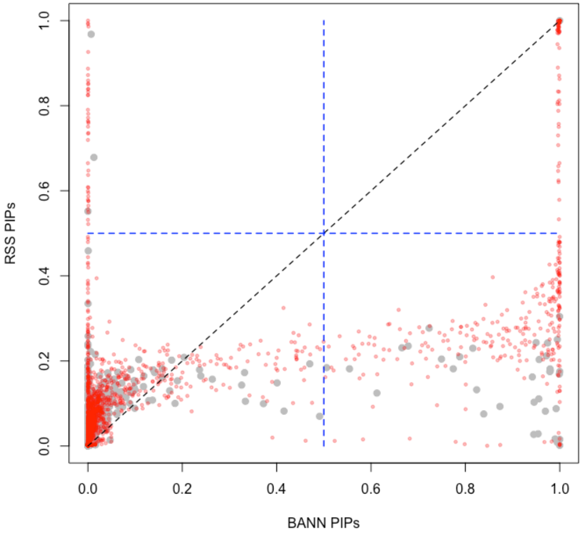

(D) SNP-Set Methods (Polygenic Traits)
