# Supplementary material for: Multi-scale inference of genetic trait architecture using biologically annotated neural networks
Source: PLoS Genet. 2021 Aug 19;17(8):e1009754. doi: 10.1371/journal.pgen.1009754 (PMC8407593; doi:10.1371/journal.pgen.1009754)
Supplement: S22 Fig — Quantitative traits are simulated to have broad-sense heritability of H2 = 0.2 with equal contributions from additive effects and epistatic interactions (i.e., ρ = 0.5). We consider two different trait architectures: (A, B) sparse where only 1% of SNP-sets are enriched for the trait; and (C, D) polygenic where 10% of SNP-sets are enriched. We set the number of causal SNPs with non-zero effects to be 1% and 10% of all SNPs located within the enriched SNP-sets, respectively. In these simulations, traits were generated while also using the top ten principal components (PCs) of the genotype matrix as covariates. Results are shown comparing the posterior inclusion probabilities (PIPs) derived by the BANNs model fit with individual-level data on the x-axis and (A, C) SuSiE [46] and (B, D) RSS [26] on the y-axis, respectively. Here, SuSie is fit while assuming a high maximum number of causal SNPs (ℓ = 3000). The blue horizontal and vertical dashed lines are marked at the “median probability criterion” (i.e., PIPs for SNPs and SNP-sets greater than 0.5) [57]. True positive causal variants used to generate the synthetic phenotypes are colored in red, while non-causal variants are given in grey. SNPs and SNP-sets in the top right quadrant are selected by both approaches; while, elements in the bottom right and top left quadrants are uniquely identified by BANNs and SuSie/RSS, respectively. Each plot combines results from 100 simulated replicates (see S1 Text). (PDF) [file pgen.1009754.s022.pdf]

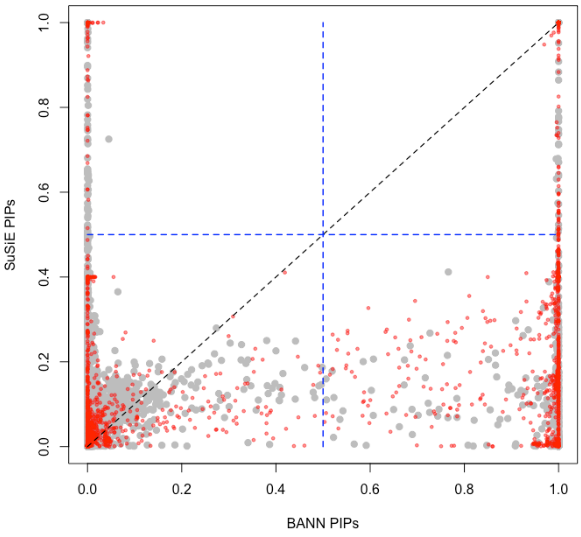

(A) SNP-Level Methods (Sparse Traits)

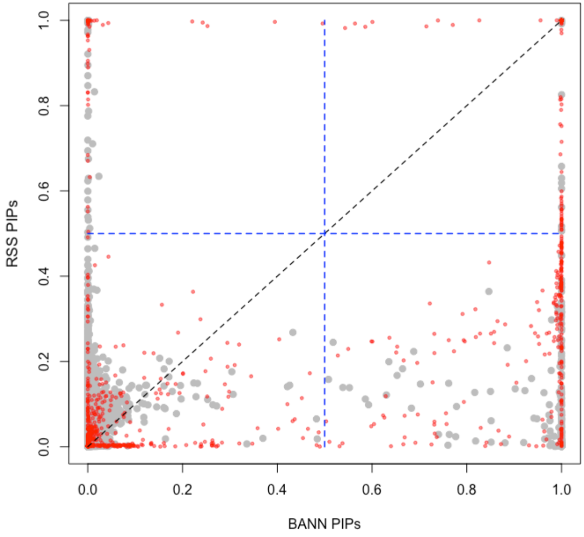

(B) SNP-Set Methods (Sparse Traits)

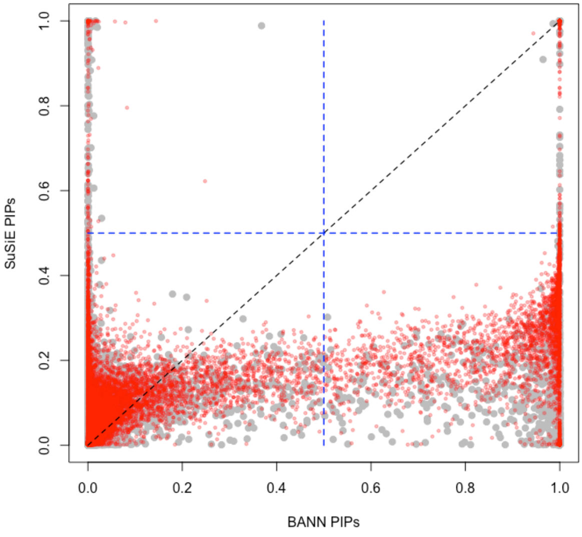

(C) SNP-Level Methods (Polygenic Traits)

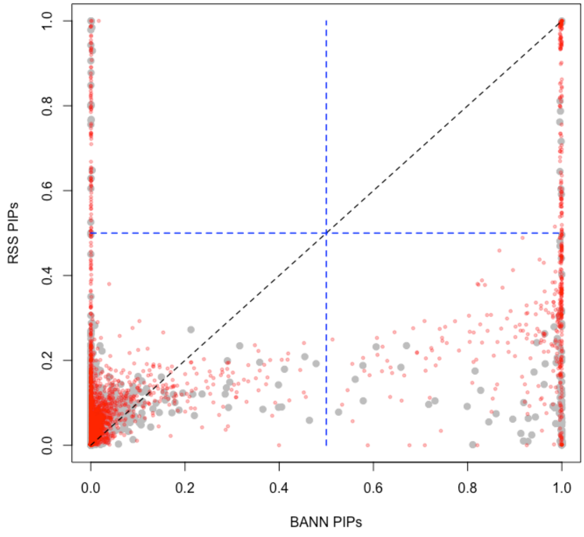

(D) SNP-Set Methods (Polygenic Traits)
