# Supplementary figures and images for: Multi-scale inference of genetic trait architecture using biologically annotated neural networks
Source: PLoS Genet. 2021 Aug 19;17(8):e1009754. doi: 10.1371/journal.pgen.1009754 (PMC8407593; doi:10.1371/journal.pgen.1009754)

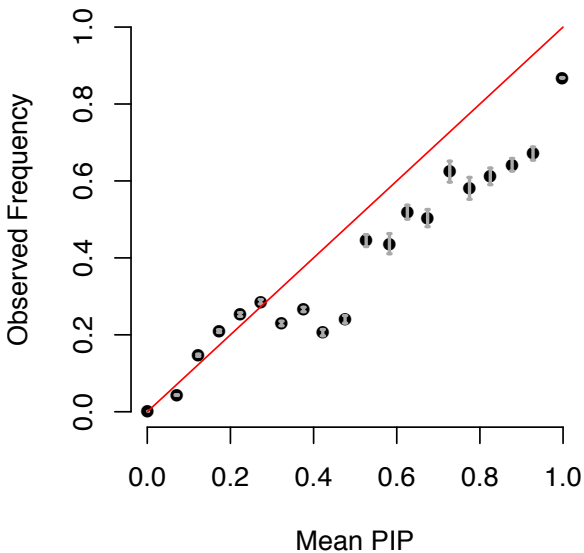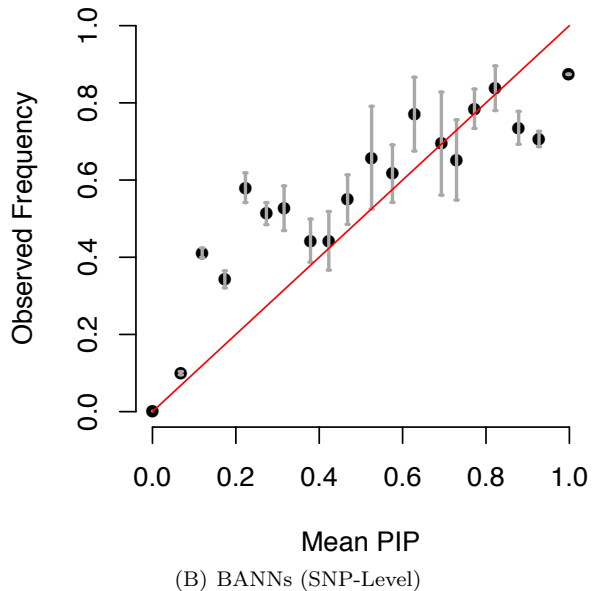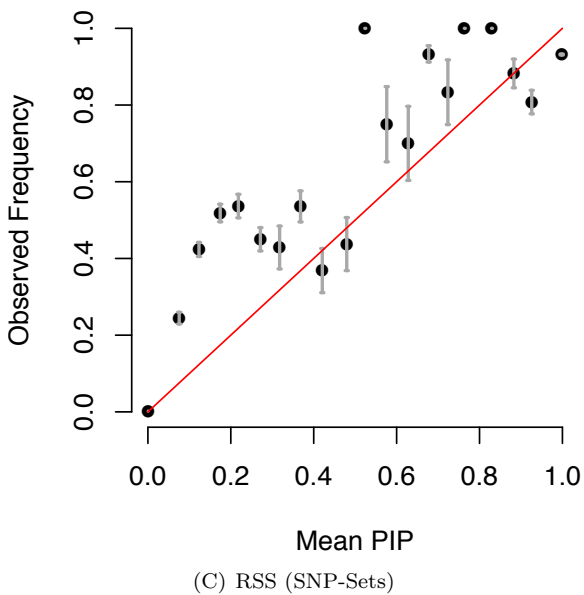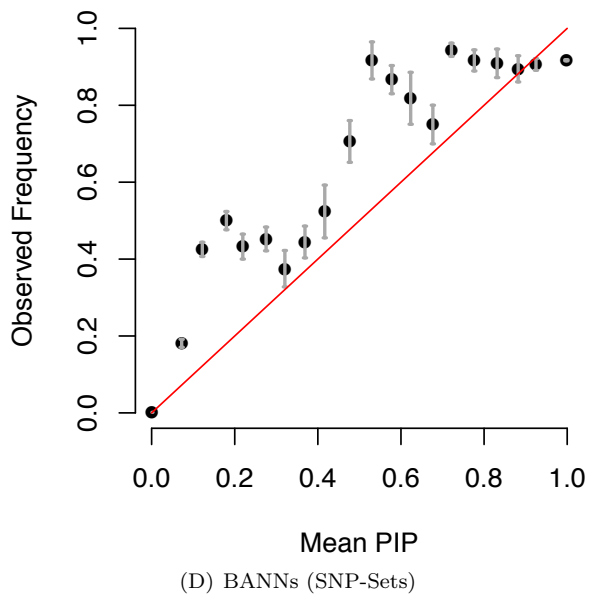

Supplement: S24 Fig — This experiment follows largely from previous work [46, 65]. Here, SNPs and SNP-sets across simulations are grouped into bins according to their reported PIPs (using 20 equally spaced bins, from 0 to 1). The plots show the average PIP for each bin against the proportion of causal SNPs or SNP-sets in that bin. A well calibrated method should produce points near the x-axis = y-axis line (i.e., the diagonal red lines). Gray error bars show ±2 standard errors. Panel (A, B) shows the comparison of BANNs SNP layer with SuSiE [46], and (C, D) shows the comparison of BANNs SNP-set layer with RSS [26]. While the inclusion probabilities are not perfectly calibrated for any of the methods, the empirical power and false discovery rate (FDR) above the “median probability criterion” (i.e., PIPs for SNPs and SNP-sets greater than 0.5) [57] are still reasonably well controlled (see S1–S8 Tables). We hypothesize that these calibration results are due both to consequences of both variational inference and the level of polygenicity with which we simulated synthetic phenotypes. (PDF) [file pgen.1009754.s024.pdf]

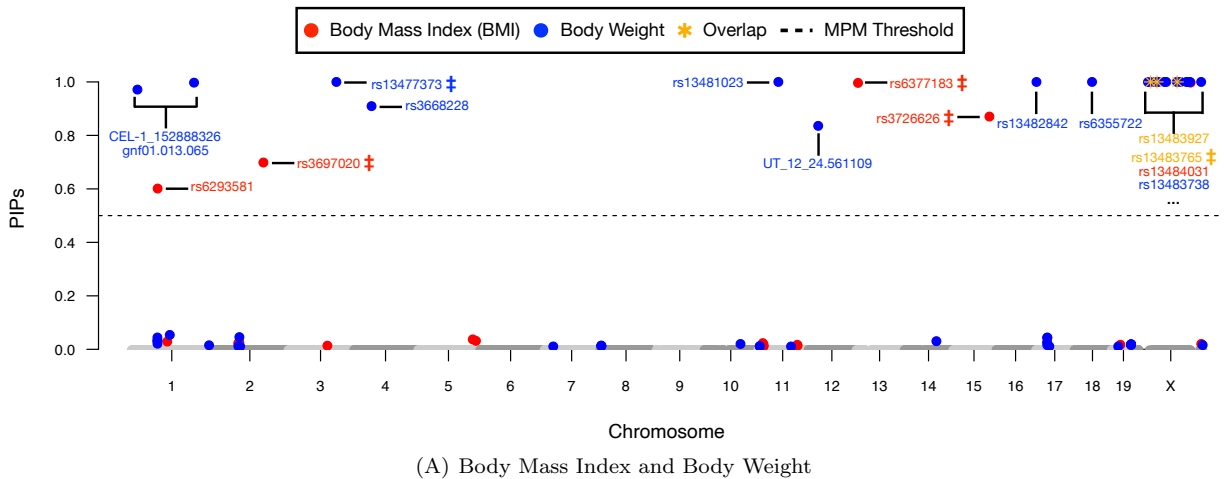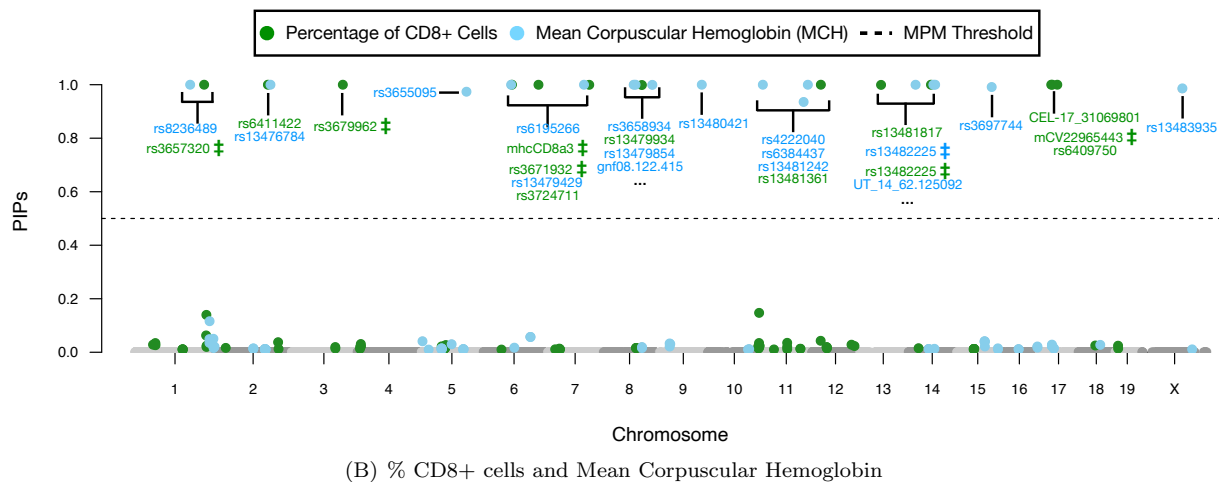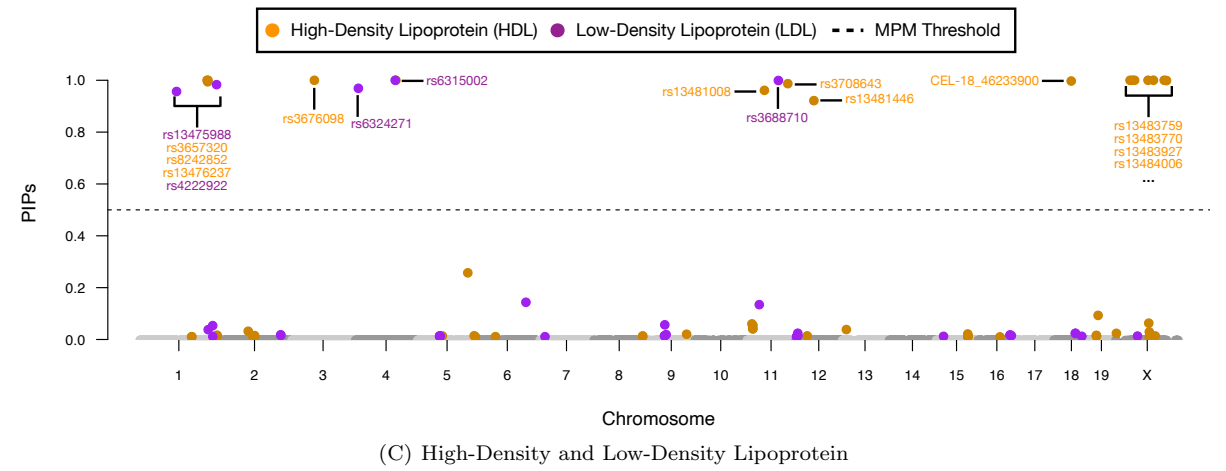

Supplement: S28 Fig — Traits are grouped based on their category and include: (A) body mass index (BMI) and body weight, (B) percentage of CD8+ cells and mean corpuscular hemoglobin (MCH), and (C) high-density and low-density lipoprotein (HDL and LDL, respectively) cholesterol. Posterior inclusion probabilities (PIP) for the input layer weights are derived from the BANNs model fit on individual-level data and are plotted for each SNP against their genomic positions. Chromosomes are shown in alternating colors for clarity. The black dashed line is marked at 0.5 and represents the “median probability model (MPM)” threshold [57]. Here, we only color code SNPs that had a PIP greater than 1% in either trait. SNPs with PIPs exceeding 1% in both traits are marked by a star and denoted as falling in the “overlap” category. BANNs estimated the following PVEs on the SNP and SNP-set levels for these traits, respectively: (i) 0.09 and 0.08 for BMI, (ii) 0.39 and 0.40 for body weight, (iii) 0.51 and 0.48 for percentage of CD8+ cells, (iv) 0.34 and 0.32 for MCH, (v) 0.34 and 0.28 for HDL, and (vi) 0.15 and 0.15 for LDL. (PDF) [file pgen.1009754.s028.pdf]

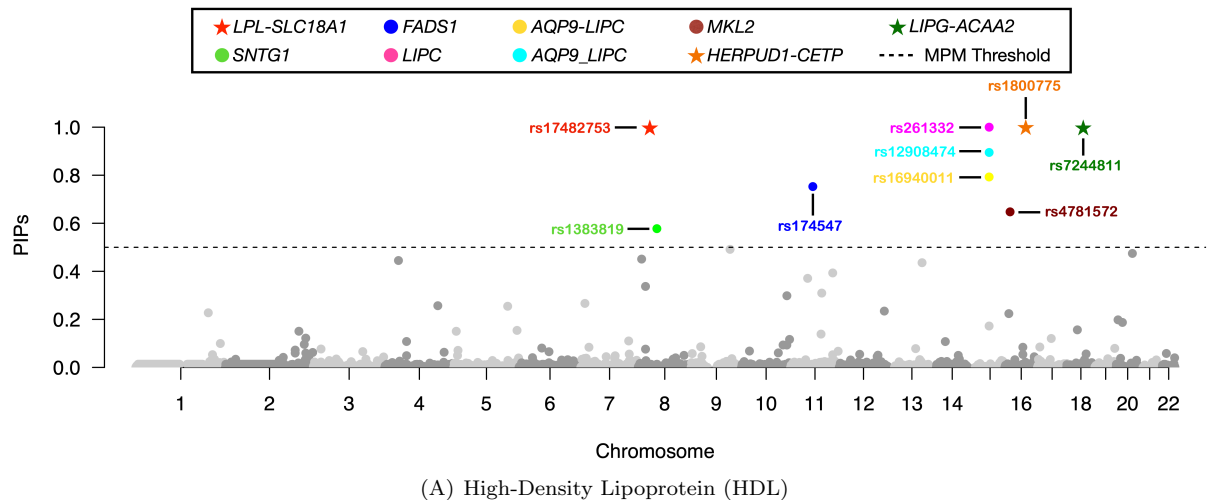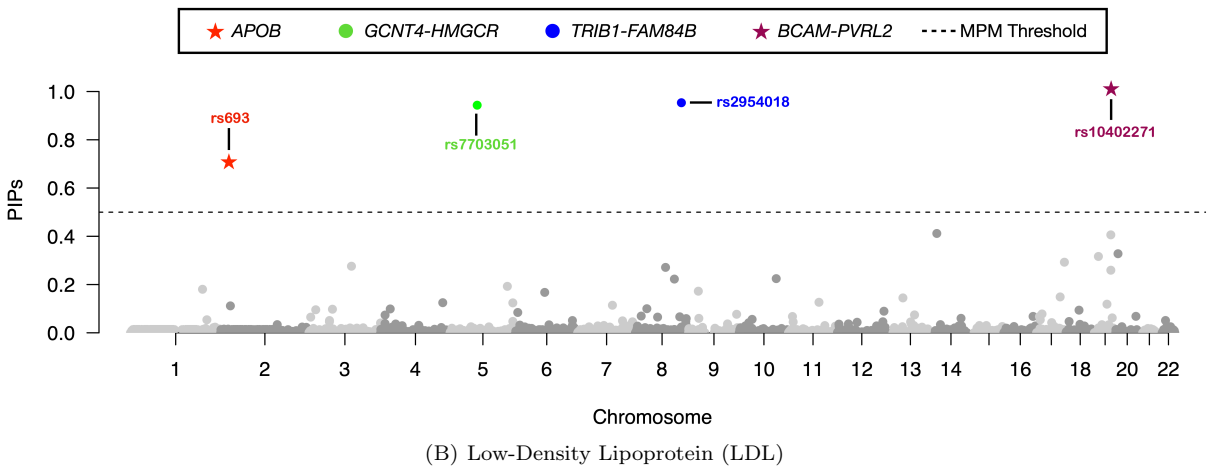

Supplement: S31 Fig — Posterior inclusion probabilities (PIP) for the neural network weights are derived from the BANNs model fit on individual-level data and are plotted for each SNP against their genomic positions. Chromosomes are shown in alternating colors for clarity. The black dashed line is marked at 0.5 and represents the “median probability model” threshold [57]. SNPs with PIPs above that threshold are color coded based on their SNP-set annotation. Here, SNP-set annotations are based on gene boundaries defined by the NCBI’s RefSeq database in the UCSC Genome Browser [50]. Unannotated SNPs located within the same genomic region were labeled as being within the “intergenic region” between two genes. These regions are labeled as Gene1-Gene2 in the legend. Gene set enrichment analyses for these SNP-sets can be found in S31 Fig. Stars (★) denote SNPs and SNP-sets that replicate findings from our analyses of HDL and LDL in the Framingham Heart Study (see Fig 4 in the main text). (PDF) [file pgen.1009754.s031.pdf]
