# Supplementary material for: Multi-scale inference of genetic trait architecture using biologically annotated neural networks
Source: PLoS Genet. 2021 Aug 19;17(8):e1009754. doi: 10.1371/journal.pgen.1009754 (PMC8407593; doi:10.1371/journal.pgen.1009754)
Supplement: S25 Fig — To investigate how choices in the model setup contribute to variable selection, we performed an “ablation analysis” where we modified parts of the BANNs framework independently and observed their direct effect on model performance (see S1 Text). We considered two different modifications to our model: (1) removing the activation function and training a fully linear hierarchical model, and (2) removing the approximate Bayesian model averaging approach and updating the probabilities πθ and πw as additional parameters in the variational EM algorithm. In the normal BANNs setup, we initialize L different models with varying priors for inclusion probabilities specified over a grid {πθ(1),…,πθ(L)}∈[1/J,1] and {πw(1),…,πw(L)}∈[1/G,1], respectively. However, in the case of the latter ablation modification, we initialize πθ = 1/J and πw = 1/G as an analogy to the “single causal variant” assumption frequently used in fine mapping [46]. Next, we update their values in the M-step of the algorithm according to the following analytic expressions: (A, C) πθ/1 − πθ = ∑j ∑k αjk/∑j ∑k(1 − αjk), and (B, D) πw/1 − πw = ∑g αg/∑g(1 − αg). Results here are shown using simulations with the self-identified “white British” ancestry cohort from the UK Biobank on synthetic traits that have broad-sense heritability H2 = 0.6 with sparse genetic architecture. Each plot combines results from 100 simulated replicates (see S1 Text). (PDF) [file pgen.1009754.s025.pdf]

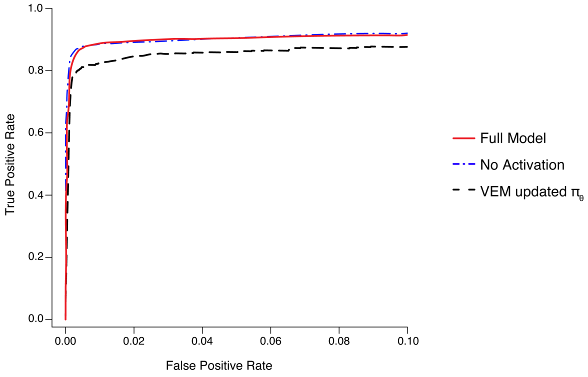

(A) SNP-Level (Additive Traits)

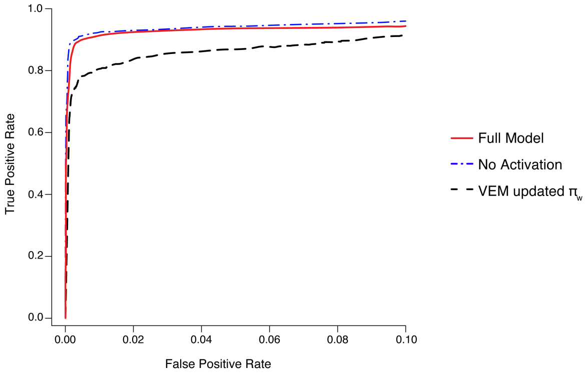

(B) SNP-Sets (Additive Traits)

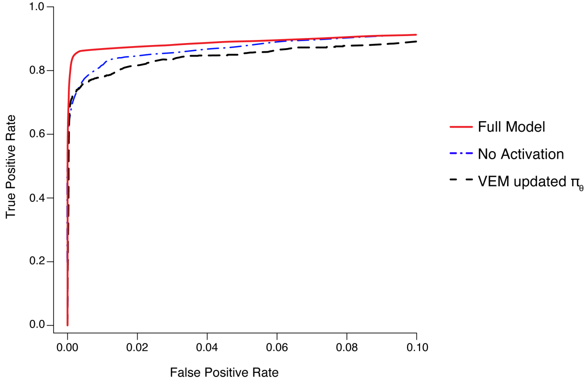

(C) SNP-Level (Additive & Epistatic Traits)

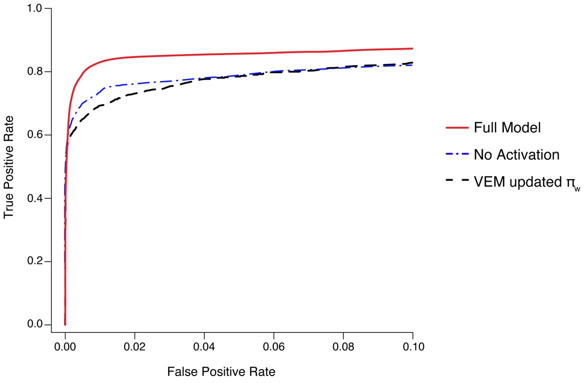

(D) SNP-Sets (Additive & Epistatic Traits)
