# Supplementary material for: Multi-scale inference of genetic trait architecture using biologically annotated neural networks
Source: PLoS Genet. 2021 Aug 19;17(8):e1009754. doi: 10.1371/journal.pgen.1009754 (PMC8407593; doi:10.1371/journal.pgen.1009754)
Supplement: S27 Fig — In this work, we define PVE as the total proportion of phenotypic variance that is explained by sparse genetic effects (both additive and non-additive) [16]. Here, quantitative traits are simulated to have broad-sense heritability of H2 = 0.6 with different levels of contributions from additive effects and epistatic interactions. We consider two different trait architectures: (A, B) sparse where only 1% of SNP-sets are enriched for the trait; and (C, D) polygenic where 10% of SNP-sets are enriched. Panels (A, C) show heritability estimates on simulations with genetic data from individuals who self-identify as being of “white British” ancestry in the UK Biobank; while, panels (B, D) show heritability estimates on simulations with genetic data from individuals who more broadly identify as being of European ancestry. True heritability values are shown as the dashed grey horizontal lines. The root mean square error (RMSE) between the BANNs model estimates of the PVE and the true values are also provided. (PDF) [file pgen.1009754.s027.pdf]

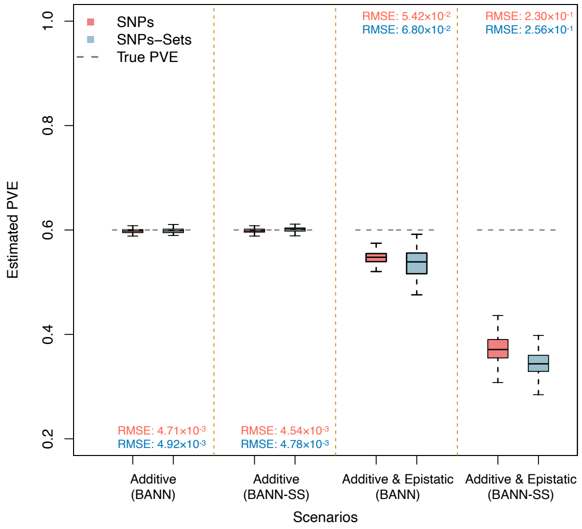

(A) Sparse Traits

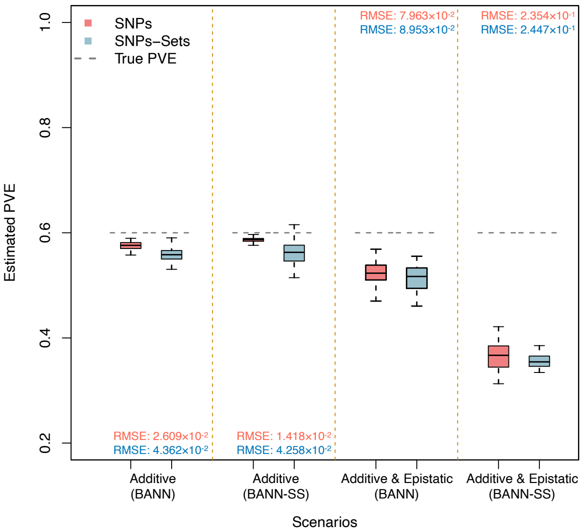

(B) Sparse Traits with Population Structure

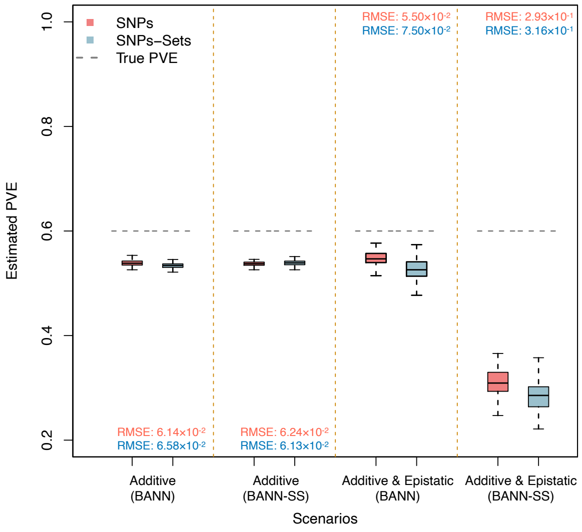

(C) Polygenic Traits

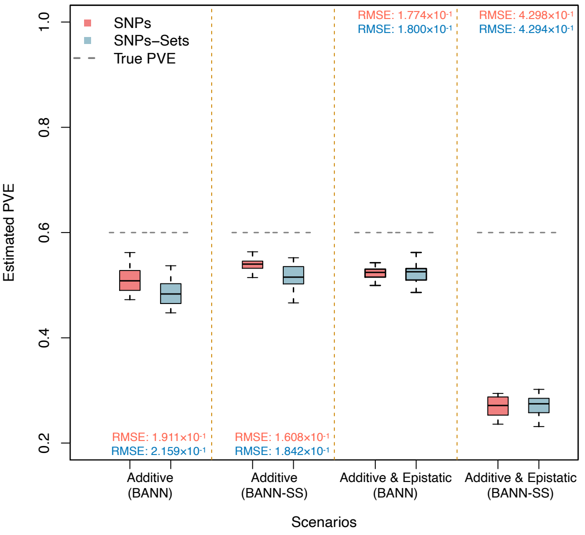

(D) Polygenic Traits with Population Structure
