# Supplementary material for: Multi-scale inference of genetic trait architecture using biologically annotated neural networks
Source: PLoS Genet. 2021 Aug 19;17(8):e1009754. doi: 10.1371/journal.pgen.1009754 (PMC8407593; doi:10.1371/journal.pgen.1009754)
Supplement: S29 Fig — Here, SNP-set annotations are based on gene boundaries defined by the NCBI’s RefSeq database in the UCSC Genome Browser [50]. Unannotated SNPs located within the same genomic region were labeled as being within the “intergenic region” between two genes. Posterior inclusion probabilities (PIP) for the input and hidden layer weights are derived by fitting the BANNs model on individual-level data. A SNP-set is considered significant if it has a PIP(g) ≥ 0.5 (i.e., the “median probability model” threshold [57]). We take these significant SNP-sets and conduct “gene set enrichment analysis” using Enrichr [90, 91] to identify the categories they overrepresent in (A, B) the database of Genotypes and Phenotypes (dbGaP) and (C, D) the GWAS Catalog (2019). Nearly all enriched categories are related with (A, C) HDL and (B, D) LDL, respectively. Note that in LDL, the BANNs framework identified the gene APOB as having a high PIP = 0.976. There have been hypotheses connecting LDL to cognitive traits [140, 141], and APOB has been shown to be related to cerebrospinal fluid and memory [142–144]. Therefore, we argue that results in panel (D) are also relevant. (PDF) [file pgen.1009754.s029.pdf]

(a)

	p value	*q* value	Odds.ratio	Combined score	# of sig. genes in dbGaP
Metabolic Syndrome X	1.316e-04	4.541e-02	115.94	1036.01	2
Lipoproteins, HDL	9.365e-04	1.616e-01	43.72	304.85	2
Cholesterol, HDL	2.188e-03	2.516e-01	11.20	68.63	3
Apolipoprotein A-I	8.221e-03	7.091e-01	121.21	581.95	1
Natriuretic Peptide, Brain	1.342e-02	9.26e-01	74.07	319.33	1
Triglycerides	1.402e-02	8.06e-01	10.93	46.64	2
Lipids	6.332e-02	1.000	15.33	42.29	1
Arteries	6.473e-02	1.000	14.98	41.01	1
Alcoholism	6.755e-02	1.000	14.34	38.64	1
Iron	1.095e-01	1.000	8.66	19.15	1

(b)

	p value	*q* value	Odds.ratio	Combined score	# of sig. genes in dbGaP
1-Alkyl-2-acetylglycerophosphocholine Esterase	2.098e-03	7.239e-01	476.19	2936.47	1
Lipoproteins, LDL	1.075e-02	1.000	92.59	419.68	1
Alzheimer Disease	2.376e-02	1.000	41.67	155.82	1
Coronary Disease	2.435e-02	1.000	40.65	151.02	1
Coronary Artery Disease	5.995e-02	1.000	16.26	45.76	1
Myocardial Infarction	6.677e-02	1.000	14.56	39.40	1
Triglycerides	7.101e-02	1.000	13.66	36.13	1
Cholesterol	7.776e-02	1.000	12.44	31.77	1
Cholesterol, LDL	8.781e-02	1.000	10.96	26.67	1
Cholesterol, HDL	1.024e-01	1.000	9.34	21.27	1

(c)

	p value	*q* value	Odds.ratio	Combined score	# of sig. genes in GWAS Catalog
Apolipoprotein A1 levels	3.409e-09	5.922e-06	800	15597.40	3
High density lipoprotein cholesterol levels	1.932e-07	1.678e-04	75.12	1161.28	4
HDL cholesterol levels	5.749e-07	3.329e-04	57.35	824.03	4
HDL cholesterol	6.234e-07	2.707e-04	28.01	400.23	5
Triglyceride levels	9.011e-07	3.130e-04	51.28	713.83	4
Metabolite levels (lipoprotein measures)	9.875e-07	2.859e-04	148.15	2048.61	3
Lipid metabolism phenotypes	2.611e-06	6.480e-04	108.11	1389.80	3
Metabolic syndrome	4.752e-06	1.032e-03	88.89	1089.51	3
Mean diameter of HDL particles	7.861e-06	1.517e-03	444.44	5223.81	2
Total cholesterol levels	9.924e-06	1.724e-03	28.07	323.38	4

(d)

	p value	*q* value	Odds.ratio	Combined score	# of sig. genes in GWAS Catalog
Waist-to-hip circumference ratio (smoking years interaction)	7.497e-07	1.302e-03	1333.33	18804.85	2
Cerebrospinal fluid t-tau levels in mild cognitive impairment	1.018e-05	8.841e-03	392.16	4507.90	2
Cerebrospinal AB1-42 levels in mild cognitive impairment	1.280e-05	7.409e-03	350.88	3953.12	2
Cerebrospinal AB1-42 levels in Alzheimer's disease dementia	1.571e-05	6.822e-03	317.46	3511.50	2
Logical memory (immediate recall)	1.892e-05	6.573e-03	289.86	3152.23	2
Logical memory (delayed recall)	2.064e-05	5.121e-03	277.78	2996.75	2
Cerebrospinal fluid p-tau levels	2.064e-05	5.975e-03	277.78	2996.75	2
Cerebrospinal fluid p-tau levels in mild cognitive impairment	2.43e-05	5.275e-03	256.41	2724.40	2
Cerebrospinal fluid t-tau levels	2.624e-05	5.064e-03	246.91	2604.53	2
Bladder cancer	2.825e-05	4.907e-03	238.10	2493.89	2
