# Supplementary material for: Multi-scale inference of genetic trait architecture using biologically annotated neural networks
Source: PLoS Genet. 2021 Aug 19;17(8):e1009754. doi: 10.1371/journal.pgen.1009754 (PMC8407593; doi:10.1371/journal.pgen.1009754)
Supplement: S30 Fig — Here, SNP-set annotations are based on gene boundaries defined by the NCBI’s RefSeq database in the UCSC Genome Browser [50]. Unannotated SNPs located within the same genomic region were labeled as being within the “intergenic region” between two genes. In this analysis, each gene boundary annotation is modfied by adding SNPs within a ±500 kilobase (kb) buffer to account for possible regulatory elements. Posterior inclusion probabilities (PIP) for the input and hidden layer weights are derived by fitting the BANNs model on individual-level data. A SNP-set is considered significant if it has a PIP(g) ≥ 0.5 (i.e., the “median probability model” threshold [57]). We take these significant SNP-sets and conduct “gene set enrichment analysis” using Enrichr [90, 91] to identify the categories they overrepresent in (A, B) the database of Genotypes and Phenotypes (dbGaP) and (C, D) the GWAS Catalog (2019). Nearly all enriched categories are related with (A, C) HDL and (B, D) LDL, respectively. (PDF) [file pgen.1009754.s030.pdf]

(a)

	p value	*q* value	Odds.ratio	Combined score	# of sig. genes in GWAS Catalog
Blood Pressure Determination	2.035e-03	4.681e-02	33.8	210	2
Natriuretic Peptide, Brain	1.874e-02	1.678e-01	58.7	234	1
Brain	3.512e-02	1.678e-01	30.2	101	1
Body Weight Changes	3.512e-02	1.678e-01	30.2	101	1
Glomerular Filtration Rate	4.622e-02	1.678e-01	22.7	69.6	1
Erythrocytes	5.323e-02	1.678e-01	19.5	57.3	1
Hypertrophy, Left Ventricular	5.721e-02	1.678e-01	18.1	51.8	1
Exercise Test	6.906e-02	1.678e-01	14.9	39.7	1
Elbow	7.297e-02	1.678e-01	14	36.7	1
Amyotrophic Lateral Sclerosis	7.688e-02	1.678e-01	13.3	34	1

(b)

	p value	*q* value	Odds.ratio	Combined score	# of sig. genes in dbGaP
Cholesterol, HDL	8.286e-03	4.142e-02	18.4	88.4	2
Blood Flow Velocity	1.035e-02	4.142e-02	114	521	1
Insulin Resistance	2.297e-02	4.571e-02	50	189	1
Lipoproteins, HDL	2.415e-02	4.571e-02	47.5	177	1
Amyotrophic Lateral Sclerosis	3e-02	4.571e-02	37.9	133	1
Lipids	3.428e-02	4.571e-02	33.1	112	1
Macular Degeneration	4.624e-02	5.285e-02	24.3	74.6	1
Iron	5.998e-02	5.998e-02	18.5	52.1	1

(c)

	p value	*q* value	Odds.ratio	Combined score	# of sig. genes in GWAS Catalog
Seborrheic dermatitis	3.495e-03	3.146e-02	370	2093	1
QRS duration	1.909e-02	6.966e-02	61.5	244	1
PR interval	2.322e-02	6.966e-02	50.3	189	1
Creatinine levels	3.346e-02	7.244e-02	34.5	117	1
QT interval	4.024e-02	7.244e-02	28.6	91.8	1
Triglycerides	6.266e-02	8.311e-02	18	50	1
Total cholesterol levels	6.464e-02	8.311e-02	17.5	47.8	1
Diastolic blood pressure	2.054e-01	2.085e-01	5	7.91	1
Systolic blood pressure	2.085e-01	2.085e-01	4.91	7.7	1

(d)

	p value	*q* value	Odds.ratio	Combined score	# of sig. genes in GWAS Catalog
Low density lipoprotein cholesterol levels	1.313e-04	5.199e-03	158	1415	2
Ischemic stroke	3.055e-04	5.199e-03	102	827	2
Stroke	3.627e-04	5.199e-03	93.5	741	2
Metabolite levels	8.363e-04	8.99e-03	60.8	431	2
HDL cholesterol change in response to fenofibrate	1.999e-03	1.719e-02	714	4437	1
Cholesterol efflux capacity	2.797e-03	2.005e-02	476	2798	1
Metabolic syndrome (bivariate traits)	3.595e-03	2.208e-02	357	2008	1
Non-small cell lung cancer	6.383e-03	3.429e-02	190	962	1
C-reactive protein levels or HDL-cholesterol levels (pleiotropy)	7.973e-03	3.429e-02	150	726	1
Cardiovascular disease risk factors	7.973e-03	3.429e-02	150	726	1
