# Supplementary material for: Multi-scale inference of genetic trait architecture using biologically annotated neural networks
Source: PLoS Genet. 2021 Aug 19;17(8):e1009754. doi: 10.1371/journal.pgen.1009754 (PMC8407593; doi:10.1371/journal.pgen.1009754)
Supplement: S32 Fig — Here, SNP-set annotations are based on gene boundaries defined by the NCBI’s RefSeq database in the UCSC Genome Browser [50]. Unannotated SNPs located within the same genomic region were labeled as being within the “intergenic region” between two genes. Posterior inclusion probabilities (PIP) for the input and hidden layer weights are derived by fitting the BANNs model on individual-level data. A SNP-set is considered significant if it has a PIP(g) ≥ 0.5 (i.e., the “median probability model” threshold [57]). We take these significant SNP-sets and conduct “gene set enrichment analysis” using Enrichr [90, 91] to identify the categories they overrepresent in (A, B) the database of Genotypes and Phenotypes (dbGaP) and (C, D) the GWAS Catalog (2019). Nearly all enriched categories are related with (A, C) HDL and (B, D) LDL, respectively. Note that in LDL, the BANNs framework again identifies the gene APOB as having a high PIP (replicating the finding in the Framingham Heart Study). There have been hypotheses connecting LDL to cognitive traits [140, 141], and APOB has been shown to be related to cerebrospinal fluid and memory [142–144]. Therefore, we argue that results in panel (B) are also relevant (a similar argument can be made for S33 Fig). (PDF) [file pgen.1009754.s032.pdf]

(a)

	p value	*q* value	Odds.ratio	Combined score	# of sig. genes in dbGaP
Lipoproteins, HDL	2.812e-10	9.701e-08	67.84	1491.82	6
Cholesterol, HDL	1.216e-09	2.098e-07	17.39	356.90	9
Metabolic Syndrome X	4.760e-06	5.474e-04	89.96	1102.42	3
Lipid Metabolism	7.262e-05	6.264e-03	153.27	1460.57	2
Electrocardiography	2.677e-04	1.847e-02	23.78	195.61	3
Triglycerides	4.038e-04	2.322e-02	11.31	88.35	4
Uric Acid	1.310e-03	6.457e-02	37.28	247.44	2
Iron	1.412e-03	6.091e-02	13.43	88.16	3
Phosphatidylcholines	4.343e-03	1.665e-01	229.89	1250.34	1
Epilepsies, Partial	5.788e-03	1.997e-01	172.41	888.28	1

(b)

	p value	*q* value	Odds.ratio	Combined score	# of sig. genes in dbGaP
Lipoproteins, LDL	3.579e-06	1.235e-03	98.04	1229.45	3
Cholesterol, HDL	1.976e-04	3.409e-02	13.18	112.43	4
Metabolic Syndrome X	1.938e-07	3.329e-04	51.15	201.72	1
Cholesterol	2.131e-02	1.000	8.78	33.79	2
Menopause	2.604e-02	1.000	37.95	138.45	1
Cholesterol, LDL	2.694e-02	1.000	7.34	27.97	2
Eosinophils	3.840e-02	1.000	25.58	83.37	1
Lipoproteins, HDL	5.062e-02	1.000	19.29	57.54	1
Alzheimer Disease	6.589e-02	1.000	14.71	40.00	1
Coronary Disease	6.749e-02	1.000	14.35	38.68	1

(c)

	p value	*q* value	Odds.ratio	Combined score	# of sig. genes in GWAS Catalog
High density lipoprotein cholesterol levels	6.787e-014	1.179e-10	77.70	2356.18	8
Metabolic syndrome	2.690e-13	2.337e-10	107.28	3105.10	7
HDL cholesterol levels	6.635e-13	3.685e-10	59.33	1666.02	8
HDL cholesterol	7.740e-13	3.361e-10	28.98	808.09	10
Metabolite levels (lipoprotein measures)	1.550e-12	5.384e-10	153.26	4167.47	6
Triglyceride levels	1.593e-12	4.612e-10	53.05	1441.12	8
Metabolite levels	2.713e-12	6.733e-10	49.70	1323.78	8
Lipid metabolism phenotypes	1.205e-11	2.617e-09	111.84	2811.73	6
C-reactive protein levels or HDL-cholesterol levels (pleiotropy)	6.803e-11	1.313e-08	172.41	4036.39	5
Cholesterol, total	3.479e-10	6.043e-08	19.90	868.93	7

(d)

	p value	*q* value	Odds.ratio	Combined score	# of sig. genes in GWAS Catalog
LDL cholesterol	5.756e-09	1.000e-05	39.00	739.90	6
Cerebrospinal AB1-42 levels in Alzheimer's disease dementia	6.72e-07	5.836e-04	168.07	2388.75	3
Metabolite levels (lipoprotein measures)	1.473e-06	8.530e-04	130.72	1755.31	3
Body mass index x age interaction	4.942e-06	2.146e-03	88.24	1078.03	3
Waist-to-hip circumference ratio (smoking years interaction)	6.790e-06	2.359e-03	470.59	5600.05	2
Total cholesterol levels	1.705e-05	4.935e-03	24.77	271.94	4
Response to ziprazidone in schizophrenia	1.898e-05	4.710e-03	294.12	3197.64	2
LDL cholesterol levels	2.058e-05	4.469e-03	55.15	595.10	3
Body mass index (age>50)	2.581e-05	4.982e-03	51.15	540.39	3
HDL cholesterol	4.120e-05	7.156e-03	19.77	199.65	4
