# Supplementary material for: Multi-scale inference of genetic trait architecture using biologically annotated neural networks
Source: PLoS Genet. 2021 Aug 19;17(8):e1009754. doi: 10.1371/journal.pgen.1009754 (PMC8407593; doi:10.1371/journal.pgen.1009754)
Supplement: S33 Fig — Here, SNP-set annotations are based on gene boundaries defined by the NCBI’s RefSeq database in the UCSC Genome Browser [50]. Unannotated SNPs located within the same genomic region were labeled as being within the “intergenic region” between two genes. In this analysis, each gene boundary annotation is modfied by adding SNPs within a ±500 kilobase (kb) buffer to account for possible regulatory elements. Posterior inclusion probabilities (PIP) for the input and hidden layer weights are derived by fitting the BANNs model on individual-level data. A SNP-set is considered significant if it has a PIP(g) ≥ 0.5 (i.e., the “median probability model” threshold [57]). We take these significant SNP-sets and conduct “gene set enrichment analysis” using Enrichr [90, 91] to identify the categories they overrepresent in (A, B) the database of Genotypes and Phenotypes (dbGaP) and (C, D) the GWAS Catalog (2019). Note that for panel (A), BANNs did not find many enriched SNP-sets with PIPs meeting the “median probability model” threshold and so we used a lower SNP-set threshold (PIP ≥ 0.1) to enable Enrichr to find associated dbGaP categories. (PDF) [file pgen.1009754.s033.pdf]

(a)

	p value	q value	Odds.ratio	Combined score	# of sig. genes in dbGaP
Neurotic Disorders	3.474e-05	1.806e-02	344.22	3534.359	2
Conduct Disorder	2.753e-04	7.157e-03	98.30	805.849	2
Respiratory Function Tests	1.624e-03	2.790e-02	14.35	92.163	3
Cholesterol, HDL	2.146e-02	2.790e-02	8.23	50.581	4
Erythrocytes	2.938e-03	3.055e-02	27.5	160	2
Myocardial Infarction	5.257e-03	3.958e-02	9.36	49.1	3
Epilepsies, Partial	6.186e-03	3.958e-02	222	1128	1
Triglycerides	6.266e-03	3.958e-02	8.77	44.5	3
Lipids	8.014e-03	3.958e-02	16.1	77.9	2
C-Reactive Protein	8.192e-03	3.958e-02	15.9	76.6	2

(b)

	p value	q value	Odds.ratio	Combined score	# of sig. genes in dbGaP
Leukocyte Count	2.374e-02	1.197e-01	46.5	174	1
Mental Competency	2.492e-02	1.197e-01	44.2	163	1
Body Weights and Measures	5.098e-02	1.197e-01	21	62.6	1
Arteries	5.213e-02	1.197e-01	20.6	60.7	1
Waist-Hip Ratio	5.441e-02	1.197e-01	19.7	57.2	1
Body Weight	1.222e-01	2.24e-01	8.36	17.6	1
Cholesterol	1.495e-01	2.289e-01	6.71	12.8	1
Cholesterol, LDL	1.679e-01	2.289e-01	5.91	10.5	1
Cholesterol, HDL	1.944e-01	2.289e-01	5.01	8.21	1
Body Height	2.081e-01	2.289e-01	4.64	7.29	1

(c)

	p value	q value	Odds.ratio	Combined score	# of sig. genes in GWAS Catalog
Immune response to measles-mumps-rubella vaccine	2.710e-06	5.308e-04	143	1827	3
Motion sickness	1.622e-05	1.59e-03	73.7	813	3
Cardiovascular risk factors (age interaction)	8.314e-05	5.087e-03	197	1848	2
Brain imaging	1.038e-04	5.087e-03	172	1578	2
Cutaneous squamous cell carcinoma	2.091e-04	8.198e-03	115	972	2
Waist-to-hip ratio adjusted for body mass index	6.678e-04	2.181e-02	19.7	144	3
HIV-1 susceptibility	2.611e-06	6.480e-04	108.11	1389.80	3
Rosacea symptom severity	7.966e-04	2.231e-02	55	393	2
Mercury levels	1.334e-03	2.778e-02	15.4	102	3
Emphysema imaging phenotypes	1.34e-03	2.778e-02	41.7	276	2

(d)

	p value	q value	Odds.ratio	Combined score	# of sig. genes in GWAS Catalog
Response to ziprazidone in schizophrenia	4.791e-03	5.936e-02	259	1386	1
Nonalcoholic fatty liver disease	7.774e-03	5.936e-02	151	735	1
Exploratory eye movement dysfunction in schizophrenia	8.965e-03	5.936e-02	130	611	1
Irritable bowel syndrome	1.431e-02	5.936e-02	78.9	335	1
Coronary artery calcified atherosclerotic plaque score in type2d	1.49e-02	5.936e-02	75.6	318	1
Colorectal or endometrial cancer	1.727e-02	5.936e-02	64.8	263	1
Response to amphetamines	1.845e-02	5.936e-02	60.5	241	1
Coronary artery calcified atherosclerotic plaque	1.904e-02	5.936e-02	58.5	232	1
Feeling miserable	2.022e-02	5.936e-02	55	214	1
Emphysema imaging phenotypes	2.139e-02	5.936e-02	51.8	199	1
