# Supplementary material for: Multi-scale inference of genetic trait architecture using biologically annotated neural networks
Source: PLoS Genet. 2021 Aug 19;17(8):e1009754. doi: 10.1371/journal.pgen.1009754 (PMC8407593; doi:10.1371/journal.pgen.1009754)
Supplement: S1 Table — Here, quantitative traits are simulated to have broad-sense heritability of H2 = 0.2 with only contributions from additive effects set (i.e., ρ = 1). We consider two different trait architectures: sparse where only 1% of SNP-sets are enriched for the trait; and polygenic where 10% of SNP-sets are enriched. We set the number of causal SNPs with non-zero effects to be 1% and 10% of all SNPs located within the enriched SNP-sets, respectively. (Top) Competing SNP-level mapping approaches include: CAVIAR [45], SuSiE [46], and FINEMAP [44]. The software for SuSiE requires an input ℓ which fixes the maximum number of causal SNPs in the model. We display results when this input number is high (ℓ = 3000) and when this input number is low (ℓ = 10). (Bottom) Competing SNP-set mapping approaches include: RSS [26], PEGASUS [25], GBJ [27], SKAT [21], GSEA [43], and MAGMA [23]. Results for the BANN, BANN-SS, and other Bayesian methods are evaluated based on the “median probability criterion” (i.e., PIPs for SNPs and SNP-sets greater than 0.5) [57]. Results for the frequentist approaches are based on Bonferroni-corrected thresholds for multiple hypothesis testing (P = 0.05/36518 = 1.37 × 10^−6^ at the SNP-level and P = 0.05/2816 = 1.78 × 10^−5^ at the SNP-set level, respectively). All results are based on 100 replicates and standard deviations of the estimates across runs are given in the parentheses. Approaches with the greatest power are bolded in purple, while methods with the lowest FDR is bolded in blue. (PDF) [file pgen.1009754.s034.pdf]

		SNP-Level Approaches					
**Trait Type**	**Metric**	**BANN**	**BANN-SS**	**SuSiE (High)**	**SuSiE (Low)**	**CAVIAR**	**FINEMAP**
**Sparse**	**Power**	**0.614 (0.106)**	0.609 (0.106)	0.608 (0.087)	0.424 (0.103)	0.597 (0.167)	0.529 (0.156)
	**FDR**	0.211(0.092)	0.210 (0.104)	**0.207 (0.052)**	0.512 (0.088)	0.331 (0.155)	0.346 (0.048)
**Polygenic**	**Power**	0.119 (0.039)	**0.120 (0.053)**	0.103 (0.021)	0.072 (0.037)	0.098 (0.032)	0.101 (0.044)
	**FDR**	0.224 (0.098)	0.227 (0.107)	**0.217 (0.034)**	0.491 (0.051)	0.244 (0.086)	0.257 (0.103)

		SNP-Set Level Approaches						
**Trait Type**	**Metric**	**BANN**	**BANN-SS**	**RSS**	**PEGASUS**	**SKAT**	**MAGMA**	**GSEA**
**Sparse**	**Power**	0.608 (0.132)	0.602 (0.161)	0.609 (0.127)	0.672 (0.118)	0.543 (0.142)	**0.737 (0.123)**	0.431 (0.138)
	**FDR**	**0.052 (0.107)**	0.067 (0.098)	0.088 (0.103)	0.514 (0.137)	0.466 (0.156)	0.571 (0.126)	0.579 (0.149)
**Polygenic**	**Power**	0.078 (0.027)	0.081 (0.041)	0.073 (0.024)	0.148 (0.032)	0.112 (0.042)	**0.152 (0.039)**	0.081 (0.031)
	**FDR**	0.166 (0.151)	0.163 (0.126)	**0.065 (0.112)**	0.179 (0.098)	0.181 (0.101)	0.191 (0.018)	0.221 (0.141)
