# Supplementary material for: Multi-scale inference of genetic trait architecture using biologically annotated neural networks
Source: PLoS Genet. 2021 Aug 19;17(8):e1009754. doi: 10.1371/journal.pgen.1009754 (PMC8407593; doi:10.1371/journal.pgen.1009754)
Supplement: S9 Table — Methods compared include: BANNs, CAVIAR [45], SuSiE [46], and FINEMAP [44]. Each table entry represents the average computation time (in seconds) it takes each approach to analyze a dataset of the size indicated. Run times were measured on an Intel i5-8259U CPU with base frequency of 2.30GHz, turbo frequency of 3.80GHz, and memory 16GB 2133 MHz LPDDR3. Here, we used 4 cores for parallelization when applicable. The software for SuSiE requires an input ℓ which fixes the maximum number of causal SNPs in the model. We display results when this input parameter is high (ℓ = 3000) and when this input parameter is low (ℓ = 10). Note that we implemented BANNs using the Python 3 version of the software, and the timing for its variational algorithm includes inference on both SNPs and SNP-sets. CAVIAR and FINEMAP are set up to work with GWA summary statistics, so their inputs (and timing) are the same irrespective of the sample size. (PDF) [file pgen.1009754.s042.pdf]

Simulation Parameters		Average Run Time (seconds)				
**SNPs**	**Samples Sizes**	**BANN**	**SuSiE (low)**	**SuSiE (high)**	**CAVIAR**	**FINEMAP**
2500	1000	3.34	1.89	4.22	8.21	56.99
	2000	6.71	2.87	8.72	8.21	56.99
	4000	10.82	8.42	13.63	8.21	56.99
5000	1000	7.42	2.49	7.12	31.48	102.58
	2000	13.21	5.04	21.84	31.48	102.58
	4000	21.34	9.45	32.81	31.48	102.58
10000	1000	31.39	3.52	52.24	118.98	145.51
	2000	127.18	10.22	159.97	118.98	145.51
	4000	318.81	22.62	754.63	118.98	145.51
