# Supplementary material for: Multi-scale inference of genetic trait architecture using biologically annotated neural networks
Source: PLoS Genet. 2021 Aug 19;17(8):e1009754. doi: 10.1371/journal.pgen.1009754 (PMC8407593; doi:10.1371/journal.pgen.1009754)
Supplement: S10 Table — Methods compared include: BANNs, RSS [26], PEGASUS [25], GBJ [27], SKAT [21], GSEA [43], and MAGMA [23]. Here, we simulated 10 datasets for each pair of parameter values (number of SNP-sets analyzed and number of SNPs within each SNP-set). Sample size was held constant at n = 10,000 individuals. Each table entry represents the average computation time (in seconds) it takes each approach to analyze a dataset of the size indicated. Run times were measured on an Intel i5-8259U CPU with base frequency of 2.30GHz, turbo frequency of 3.80GHz, and memory 16GB 2133 MHz LPDDR3. Here, we used 4 cores for parallelization when applicable. Note that PEGASUS, GBJ, SKAT, and MAGMA are score-based methods and, thus, are expected to take the least amount of time to run. Both the BANNs framework and RSS are regression-based methods. The increased computational burden of these approaches results from its need to do (approximate) Bayesian posterior inference; however, the sparse and partially connected architecture of the BANNs model allows it to scale more favorably for larger dimensional datasets. Note that we implemented BANNs using the Python 3 version of the software, and the timing for its variational algorithm includes inference on both SNPs and SNP-sets. (PDF) [file pgen.1009754.s043.pdf]

Simulation Parameters		Average Run Time (seconds)						
**SNP-Sets**	**SNPs per SNP-set**	**BANN**	**RSS**	**PEGASUS**	**GBJ**	**SKAT**	**MAGMA**	**GSEA**
250	10	12.58	13.12	2.41	2.68	2.13	0.03	2.48
	20	44.32	58.21	2.13	5.18	3.82	0.08	4.68
	40	189.44	224.62	2.22	9.64	6.47	0.18	8.51
500	10	48.92	59.31	5.11	5.37	5.23	0.09	5.31
	20	223.14	244.07	5.02	11.26	9.22	0.21	11.12
	40	965.48	1026.12	5.72	27.91	14.84	0.24	20.36
1000	10	194.62	249.57	8.67	12.27	11.31	0.72	11.41
	20	1213.19	2176.33	8.93	27.62	18.16	1.48	24.93
	40	6823.31	14495.72	10.21	61.37	30.83	4.26	60.82
