# Supplementary material for: Multi-scale inference of genetic trait architecture using biologically annotated neural networks
Source: PLoS Genet. 2021 Aug 19;17(8):e1009754. doi: 10.1371/journal.pgen.1009754 (PMC8407593; doi:10.1371/journal.pgen.1009754)
Supplement: S17 Table — Here, SNP-set annotations are based on gene boundaries defined by the NCBI’s RefSeq database in the UCSC Genome Browser [50]. Unannotated SNPs located within the same genomic region were labeled as being within the “intergenic region” between two genes. These regions are labeled as Gene1-Gene2 in the table. Posterior inclusion probabilities (PIP) for the input and hidden layer weights are derived by fitting the BANNs model on individual-level data. A SNP-set is considered enriched if it has a PIP(g) ≥ 0.5 (i.e., the “median probability model” threshold [57]). We report the “top” associated SNP within each region and its corresponding PIP(j). We also report the corresponding SNP and SNP-set level results after running SuSiE [46] and RSS [26] on these same traits, respectively. The last column details references and literature sources that have previously suggested some level of association or enrichment between the each genomic region and the traits of interest. See S18 and S19 Tables for the complete list of SNP and SNP-set level results. ♣: SNPs and SNP-sets replicated in an independent analysis of ten thousand randomly sampled individuals of European ancestry from the UK Biobank [31]. (PDF) [file pgen.1009754.s050.pdf]

Trait	SNP-Set	Chr	PIP(g)	Rank	RSS PIP	RSS Rank	Top SNP	PIP(j)	SuSiE PIP	SuSiE Rank	Ref(s)
HDL	*C10rf213-TCEA3*	1	0.998	2	0.121	162	rs176141	0.298	0.120	19	[78, 79]
	ST18-FAM150A	8	0.9169	4	0.959	4	rs11990693	0.999	0.277	8	[80]
	LPL-SLC18A1	8	0.734	5	0.999	2	rs17482753$^{\clubsuit}$	0.999	0.191	11	[81, 82]
LDL	*AQP9-LIPC*	15	0.913	1	0.198	29	rs4775041	0.422	0.238	4	[83, 84]
	TRMU-CELSR1	22	0.678	2	0.895	3	rs5768939	0.379	0.185	5	[85–88]
	C10orf92-C10orf93	10	0.537	3	0.125	157	rs10781583	0.215	0.0377	26	—
