# Supplementary material for: Multi-scale inference of genetic trait architecture using biologically annotated neural networks
Source: PLoS Genet. 2021 Aug 19;17(8):e1009754. doi: 10.1371/journal.pgen.1009754 (PMC8407593; doi:10.1371/journal.pgen.1009754)
Supplement: S1 Text — Specifically, we give description of the variational inference algorithm for the BANN framework, the data quality control procedures, simulation setup and scenarios, and additional results for the traits analyzed from the Framingham Heart Study and the UK Biobank. (PDF) [file pgen.1009754.s056.pdf]

Supporting Information to "Multi-scale Inference of Genetic Trait Architecture using Biologically Annotated Neural Networks"

Pinar Demetci[1,2*], Wei Cheng[2,3*], Gregory Darnell[2], Xiang Zhou[4,5], Sohini Ramachandran[1-3], and Lorin Crawford[2,6,7†]

1 Department of Computer Science, Brown University, Providence, Rhode Island, USA
2 Center for Computational Molecular Biology, Brown University, Providence, Rhode Island, USA
3 Department of Ecology and Evolutionary Biology, Brown University, Providence, Rhode Island, USA
4 Department of Biostatistics, University of Michigan, Ann Arbor, Michigan, USA
5 Center for Statistical Genetics, University of Michigan, Ann Arbor, Michigan, USA
6 Microsoft Research New England, Cambridge, Massachusetts, USA
7 Department of Biostatistics, Brown University, Providence, Rhode Island, USA

*** Authors Contributed Equally**
† Corresponding E-mail: lcrawford@microsoft.com

Contents

1 Overview of Partially Connected Bayesian Neural Networks **2**

2 Variational Expectation-Maximization (EM) Algorithm **3**

2.1 Input Layer (SNP-Level) Updates 4

2.2 Outer Layer (SNP-Set Level) Updates 6

3 Accounting for Non-Additive Genetic Effects **7**

4 Estimating Phenotypic Variance Explained (PVE) **8**

5 Ablation Test and Analysis **8**

6 Data Quality Control Procedures for Stock of Mice **9**

7 Data Quality Control Procedures for Framingham Heart Study **9**

8 Data Quality Control Procedures for UK Biobank **10**

9 Simulation Setup and Scenarios **10**

10 Software Details **11**

11 Pseudocode for Biologically Annotated Neural Networks **13**

References **15**

1 Overview of Partially Connected Bayesian Neural Networks

Biologically annotated neural networks (BANNs) are feedforward Bayesian models with have partially connected architectures that are inspired by the hierarchical nature of biological enrichment analyses in GWA studies. The BANNs software takes in one of two data types from genome-wide association (GWA) studies: (i) individual-level data $\mathcal{D} = \{\mathbf{X}, \mathbf{y}\}$ where $\mathbf{X}$ is an $N \times J$ matrix of genotypes with J denoting the number of single nucleotide polymorphisms (SNPs) encoded as $\{0, 1, 2\}$ copies of a reference allele at each locus and $\mathbf{y}$ is an N-dimensional vector of quantitative traits (see Fig 1 in the main text); or (ii) GWA summary statistics $\mathcal{D} = \{\mathbf{R}, \widehat{\boldsymbol{\theta}}\}$ where $\mathbf{R}$ is a $J \times J$ empirical linkage disequilibrium (LD) matrix of pairwise correlations between SNPs and $\widehat{\boldsymbol{\theta}}$ are marginal effect size estimates for each SNP computed using ordinary least squares (OLS) (see S1 Fig). In either setting, the BANNs software also requires a predefined list of SNP-set annotations $\{\mathcal{S}_1, \ldots, \mathcal{S}_G\}$ to construct partially connected network layers that represent different scales of genomic units. In this section, we review the hierarchical probabilistic specification of the BANNs framework for individual data; however, note that extensions to summary statistics is straightforward and only requires substituting the genotypes $\mathbf{X}$ for the LD matrix $\mathbf{R}$ and substituting the phenotypes $\mathbf{y}$ for the OLS effect sizes $\widehat{\boldsymbol{\theta}}$.

Without loss of generality, let SNP-set g represent an annotated collection of SNPs $j \in \mathcal{S}_g$ with cardinality $|\mathcal{S}_g|$. The BANNs framework is probabilistically represented as a nonlinear regression model

$$\mathbf{y} = \sum_{g=1}^{G} h(\mathbf{X}_g \boldsymbol{\theta}_g + \mathbf{1} b_g^{(1)}) w_g + \mathbf{1} b^{(2)}, \tag{1}$$

where $\mathbf{X}_g = [\mathbf{x}_1, \ldots, \mathbf{x}_{|\mathcal{S}_g|}]$ is the subset of SNPs annotated for SNP-set g; $\boldsymbol{\theta}_g = (\theta_1, \ldots, \theta_{|\mathcal{S}_g|})$ are the corresponding inner layer weights; $h(\bullet)$ denotes the nonlinear activations defined for neurons in the hidden layer; $\mathbf{w} = (w_1, \ldots, w_G)$ are the weights for the G-predefined SNP-sets in the hidden layer; $\mathbf{b}^{(1)} = (b_1^{(1)}, \ldots, b_G^{(1)})$ and $b^{(2)}$ are deterministic biases that are produced during the network training phase in the input and hidden layers, respectively; and $\mathbf{1}$ is an N-dimensional vector of ones. For convenience, we assume that the genotype matrix (column-wise) and trait of interest have been mean-centered and standardized. In the main text, $h(\bullet)$ is defined as a Leaky rectified linear unit (Leaky ReLU) activation function [1], where $h(\boldsymbol{x}) = \boldsymbol{x}$ if $\boldsymbol{x} > 0$ and $0.01\boldsymbol{x}$ otherwise. Throughout this Supporting Information, we will equivalently write Eq. (1) in matrix notation as

$$\mathbf{y} = \mathbf{H}(\boldsymbol{\theta})\mathbf{w} + \mathbf{1} b^{(2)},$$

where $\mathbf{H}(\boldsymbol{\theta}) = [h(\mathbf{X}_1\boldsymbol{\theta}_1 + \mathbf{1}b_1^{(1)}), \ldots, h(\mathbf{X}_G\boldsymbol{\theta}_G + \mathbf{1}b_G^{(1)})]$ denotes the matrix of nonlinear neurons in the hidden layer which are empirically computed given estimates the input layer weights. The hierarchical structure of the joint likelihood can be seen as a nonlinear take on classical integrative and structural regression models frequently used in GWA analyses [2–8].

As explained in the main text, we treat the weights of the input ($\boldsymbol{\theta}$) and hidden layers ($\mathbf{w}$) as random variables which allows for multi-scale genomic inference on both SNPs and SNP-sets, simultaneously. We assume that SNP-level effects follow a sparse K-mixture of normal distributions

$$\theta_j \sim \pi_\theta \sum_{k=1}^{K} \eta_{\theta k} \mathcal{N}(0, \sigma_{\theta k}^2) + (1 - \pi_\theta)\delta_0 \tag{2}$$

where δ_0 is a point mass at zero; $\boldsymbol{\sigma}_\theta^2 = (\sigma_{\theta 1}^2, \ldots, \sigma_{\theta K}^2)$ are variance of the K nonzero mixture components; $\boldsymbol{\eta}_\theta = (\eta_{\theta 1}, \ldots, \eta_{\theta K})$ represents the marginal (unconditional) probability that a randomly selected SNP belongs to the k-th mixture component such that $\sum_k \eta_{\theta k} = 1$; and π_θ denotes the total proportion of SNPs that have a nonzero effect on the trait of interest. Notice that we write the mixture prior in this

form as a way to simplify updates in the algorithm for posterior inference. For reference, one can think of the delta mass as a normal distribution with fixed variance set to zero. Intuitively, specifying a larger K allows the neural network to learn general SNP effect size distributions spanning over a diverse class of trait architectures. For example, one can take a nonparametric approach and allow $K \to \infty$ such that Eq. (2) mirrors a Dirichlet process Gaussian mixture [9]. For results in the main text, we follow previous work and fix $K = 3$ [10–12]. This corresponds to the general hypothesis that SNPs can have large, moderate, and small nonzero effects on phenotypic variation [13]. For inference on the hidden layer, we assume that enriched SNP-sets contain at least one SNP with a nonzero effect. This simpler criterion is formulated by placing a spike and slab prior on the hidden weights

$$w_g \sim \pi_w \mathcal{N}(0, \sigma_w^2) + (1 - \pi_w)\delta_0. \tag{3}$$

where, due to the integrative form of the likelihood in Eq. (1), the magnitude of association for a SNP-set will be directly influenced by the effect size distribution of the SNPs it contains.

For the hyper-parameters in the model, we assume the following prior distributions

$$\log(\pi_\theta) \sim \mathcal{U}(-\log(J), \log(1)), \qquad \log(\pi_w) \sim \mathcal{U}(-\log(G), \log(1)). \tag{4}$$

Following previous work, relatively uninformative uniform priors are assumed over $\log \pi_\theta$ and $\log \pi_w$ to reflect our lack of knowledge *a priori* about the proportion on associated SNP and SNP-sets with nonzero weights [14–16]. To facilitate posterior computation and interpretable inference, we also introduce two vectors of binary indicator variables $\boldsymbol{\gamma}_\theta = (\gamma_{\theta 1}, \ldots, \gamma_{\theta J}) \in \{0,1\}^J$ and $\boldsymbol{\gamma}_w = (\gamma_{w1}, \ldots, \gamma_{wG}) \in \{0,1\}^G$ where we implicitly assume *a priori* that

$$\Pr[\gamma_{\theta j} = 1] = \Pr[\theta_j \neq 0] = \pi_\theta, \qquad \Pr[\gamma_{wg} = 1] = \Pr[w_g \neq 0] = \pi_w. \tag{5}$$

Alternatively, we say $\gamma_{\theta j}$ and γ_{wg} take values of 1 when weights θ_j and w_g are drawn from the normal "slabs" of Eqs. (2) and (3), respectively; they take values of 0 otherwise. In the main text, we refer to these indicators as inclusion probabilities [17] and we use the marginal posterior means of these quantities as general summaries of evidence that SNPs and SNP-sets are statistically associated with phenotypic variation.

2 Variational Expectation-Maximization (EM) Algorithm

We modify a previously developed variational expectation-maximization (EM) algorithm to estimate the posterior distribution of parameters in the BANNs framework. The derivations in this section largely follow those developed in previous work [7, 9, 18–20]. As mentioned in the main text, the overall goal of variational inference is to approximate the true posterior distribution for network parameters with a similar distribution from an approximating family [21–25]. The EM algorithm we use aims to minimize the Kullback-Leibler divergence between the exact and approximate posterior distributions, respectively. To begin, we assign exchangeable uniform hyper-priors over a grid of values on the log-scale for π_θ and π_w [15]. We then run the EM algorithm while iterating through each combination of these values. In the E-step, we use co-ordinate ascent to update the free parameters of the approximate variational posterior. In the M-step, we derive updates for the model hyper-parameters by solving for the roots of their gradients. Finally, in the last step, we empirically compute (approximate) posterior values for the network connection weights $(\boldsymbol{\theta}, \mathbf{w})$ and their corresponding inclusion probabilities by marginalizing over the different model combinations for π_θ and π_w with normalized importance weights [19, 20]. A complete overview of the algorithm is given below. Again, note that extensions to summary statistics is straightforward and only requires substituting the genotypes $\mathbf{X}$ for an LD matrix $\mathbf{R}$ and substituting the phenotypes $\mathbf{y}$ for estimated OLS effect sizes $\widehat{\boldsymbol{\theta}}$ (see Material and Methods in the main text).

Given the formulation of the BANNs model and the partially connected neural network architecture, the weights in the second layer are conditionally independent of the weights in the input layer given the activations (or outputs) from the first layer. This means that we can break up the model fitting procedure into two integrative parts and assess two different lower bounds for the input and hidden layer weights, respectively, to ensure convergence. Specifically estimates on the SNP-level are first maximized with respect to the trait of interest; while, parameters corresponding to the SNP-set level are maximized with respect to the observed trait. The software code iterates between the "inner" lower bound and the "outer" lower bound each step of the algorithm until convergence. Iterations in the algorithm are terminated when either one of two stopping criteria are met: (i) the difference between the lower bound of two consecutive updates are within some small range (specified by tolerance argument ϵ), or (ii) a maximum number of iterations is reached. For the simulations and real data analyses ran in this paper, we set $\epsilon = 1 \times 10^{-4}$ for the first criterion and used a maximum of 10,000 iterations for the second.

2.1 Input Layer (SNP-Level) Updates

For the SNP-level effects in the input layer, we aim to find a distribution $q(\boldsymbol{\theta}, \boldsymbol{\gamma}_\theta)$ that approximates the true posterior distribution $p(\boldsymbol{\theta}, \boldsymbol{\gamma}_\theta \,|\, \mathcal{D})$, where $\boldsymbol{\theta} = (\boldsymbol{\theta}_1, \ldots, \boldsymbol{\theta}_G)$ and $\mathcal{D}$ is used to denote the individual-level data and all relevant hyper-parameters. The similarity between these two distributions is maximized by minimizing the Kullback-Leibler (KL) divergence between them. This is formulated by

$$\mathrm{KL}(q(\boldsymbol{\theta}, \boldsymbol{\gamma}_\theta) \,\|\, p(\boldsymbol{\theta}, \boldsymbol{\gamma}_\theta \,|\, \mathcal{D})) = \int \log\left[\frac{q(\boldsymbol{\theta}, \boldsymbol{\gamma}_\theta)}{p(\boldsymbol{\theta}, \boldsymbol{\gamma}_\theta \,|\, \mathcal{D})}\right] q(\boldsymbol{\theta}, \boldsymbol{\gamma}_\theta)\, \mathrm{d}\boldsymbol{\theta}\, \mathrm{d}\boldsymbol{\gamma}_\theta. \tag{6}$$

Once again, to facilitate posterior inference on this layer, we follow previous work [9] by introducing another vector of binary indicator variables $\varphi_{jk} \in \{0, 1\}$ to indicate which of the K-normal components θ_j belongs to in the prior specified in Eq. (2) such that $\Pr[\varphi_{jk} = 1] = \eta_{\theta k}$. This leads to the following natural expression for the variational mixture distribution on each of the individual SNP-level weights

$$q(\theta_j, \gamma_{\theta j}; \phi_j) = \begin{cases} \sum_{k=1}^K \alpha_{jk}\, \mathcal{N}(m_{jk}, s_{jk}^2) & \text{if} \quad \gamma_{\theta j} = 1 \\ (1 - \sum_{k=1}^K \alpha_{jk})\, \delta_0 & \text{if} \quad \gamma_{\theta j} = 0 \end{cases} \tag{7}$$

where, in addition to previous notation, we assume $q[\varphi_{jk} = 1] = \alpha_{jk}$ for the binary indicators. Additionally, we will let $\phi_j = \{\alpha_{jk}, m_{jk}, s_{jk}^2\}_{k=1}^K$ denote a collection of free parameters and $\boldsymbol{\phi} = (\phi_1, \ldots, \phi_J)$ will be used to compute the approximations [26, 27]. The basic idea behind the variational approximation is to formulate a lower bound to the marginal likelihood, then to iteratively adjust the free parameters in $\boldsymbol{\phi}$ so that this bound becomes as tight as possible [19, 20, 25]. Finding the "best" factorized variational distribution amounts to finding the free parameters $\boldsymbol{\phi}$ that make the Kullback-Leibler divergence in Eq. (6) as small as possible. Taking moments with respect to the specific class of variational distributions in Eq. (7) yields the following analytical expression for the lower bound on the inner layer (or SNP-level)

$$\begin{aligned} \mathrm{LB}(\pi_\theta, \boldsymbol{\sigma}_\theta^2, \tau_\theta^2) = &-\frac{N}{2}\log(2\pi\tau_\theta^2) - \frac{1}{2\tau_\theta^2}\|\mathbf{y} - \mathbf{X}\boldsymbol{\beta}_\theta\|_2^2 - \frac{1}{2}\sum_{j=1}^J (\mathbf{X}^\intercal\mathbf{X})_{jj}\mathbb{V}[\theta_j] \\ &- \sum_{j=1}^J\sum_{k=1}^K \alpha_{jk}\log\left(\frac{\alpha_{jk}}{\pi_\theta}\right) + \frac{1}{2}\sum_{j=1}^J\sum_{k=1}^K \alpha_{jk}\left[1 + \log\left(\frac{s_{jk}^2}{\tau_\theta^2\sigma_{\theta k}^2}\right) - \frac{s_{jk}^2 + m_{jk}^2}{\tau_\theta^2\sigma_{\theta k}^2}\right] \end{aligned} \tag{8}$$

where $\tau_\theta^2 \approx \mathbb{V}[\mathbf{y} - \mathbf{X}\boldsymbol{\beta}_\theta]$ approximates the variance of residual training error in the input layer; $\|\bullet\|_2$ is the Euclidean norm; $\boldsymbol{\beta}_\theta$ is a J-dimensional estimate of the posterior mean for $\boldsymbol{\theta}$ with individual elements $\beta_{\theta j} = \sum_{k=1}^K \alpha_{jk} m_{jk}$; the term $(\mathbf{X}^\intercal\mathbf{X})_{jj}$ is the j-th diagonal component of the matrix $(\mathbf{X}^\intercal\mathbf{X})$; and $\mathbb{V}[\theta_j] = \sum_{k=1}^K \alpha_{jk}(m_{jk}^2 + s_{jk}^2) - (\sum_{k=1}^K \alpha_{jk} m_{jk})^2$ is the variance of the j-th weight under the approximating

distribution in Eq. (7). We now describe the expectation and maximization steps of the approximate EM algorithm below (see Software Details in Supporting Information, Section 10). Again, the derivations in this section largely follow those developed in previous work and other theoretical derivations can be found in those corresponding sources [7, 9, 18–20].

1. **E-Step: Update the Variational Free Parameters.** As done in Carbonetto and Stephens (2012) [19], for the E-step of the algorithm, we take partial derivatives of the variational lower bound in Eq. (8) with respect to the free parameters and conditioned on hyperparameters $(\pi_\theta, \boldsymbol{\sigma}^2_\theta, \tau^2_\theta)$. Next, we set these partial derivatives to zero and solve for m_{jk}, s^2_{jk}, and α_{jk} which yields

$$m_{jk} = \frac{s^2_{jk}}{\tau^2_\theta}\left[(\mathbf{X}^\intercal\mathbf{y})_j - \sum_{l\neq j}(\mathbf{X}^\intercal\mathbf{X})_{jl}\beta_{\theta l}\right] \tag{9}$$

$$s^2_{jk} = \tau^2_\theta\left[(\mathbf{X}^\intercal\mathbf{X})_{jj} + \frac{1}{\sigma^2_{\theta k}}\right]^{-1} \tag{10}$$

$$\alpha_{jk} = \text{Sigmoid}\left(\log\left(\frac{\pi_\theta}{1-\pi_\theta}\right) + \log\left(\frac{s_{jk}}{\sigma_{\theta k}\tau_\theta}\right) + \frac{m^2_{jk}}{2s^2_{jk}}\right) \tag{11}$$

where $\sum_k \alpha_{jk} \approx \Pr[\gamma_{\theta j} = 1 \,|\, \mathbf{y}, \mathbf{X}, \pi_\theta, \boldsymbol{\sigma}^2_\theta, \tau^2_\theta]$ and the sigmoid function is set to be the standard logistic function. Intuitively, the E-step of the algorithm for the input layer produces a collection of $\boldsymbol{\alpha}_j = (\alpha_{j1}, \ldots, \alpha_{jK})$ values to determine whether each SNP has a nonzero effect on the phenotypic variance.

2. **M-Step: Update the Variance Hyper-Parameters.** In the M-step of the algorithm, we fix the newly estimated values of the variational free parameters $\boldsymbol{\phi}$ from the E-step and maximize the lower bound in Eq. (8) with respect to $\sigma^2_{\theta k}$ and τ^2_θ. This done in the usual way of solving for roots $\sigma^2_{\theta k}$ and τ^2_θ of the gradient which yields [20]

$$\sigma^2_{\theta k} = \left[\sum_{j=1}^{J}\alpha_{jk}(m^2_{jk} + s^2_{jk})\right] \Big/ \left(\tau^2_\theta\sum_{j=1}^{J}\alpha_{jk}\right) \tag{12}$$

$$\tau^2_\theta = \left(N + \sum_{j=1}^{J}\sum_{k=1}^{K}\alpha_{jk}\right)^{-1}\left[\|\mathbf{y} - \mathbf{X}\boldsymbol{\beta}_\theta\|^2_2 + \sum_{j=1}^{J}(\mathbf{X}^\intercal\mathbf{X})_{jj}\mathbb{V}[\theta_j] + \sum_{j=1}^{J}\sum_{k=1}^{K}\frac{\alpha_{jk}}{\sigma^2_{\theta k}}(m^2_{jk} + s^2_{jk})\right] \tag{13}$$

where N is equal to the dimensionality of the trait vector (i.e., the sample size when modeling individual-level data).

Following previous work [7,14–16,19,20], we incorporate Eq. (4) and account for our lack of *a priori* knowledge about the "correct" proportion of associated SNPs with nonzero effects by placing an exchangeable uniform hyper-prior distribution over an L-valued grid of possible values where $\{\pi^{(1)}_\theta, \ldots, \pi^{(L)}_\theta\} \in [1/J, 1]$. We then use the lower bound to the likelihood in Eq. (8) to approximate the posterior distribution of π_θ. Formally, we approximate $\Pr[\pi_\theta = \pi^{(l)}_\theta \,|\, \mathbf{y}, \mathbf{X}]$ with the normalized importance weights

$$\lambda^{(l)}_\theta = \frac{\text{LB}(\pi^{(l)}_\theta, \boldsymbol{\sigma}^2_\theta, \tau^2_\theta)}{\sum_{l'=1}^{L}\text{LB}(\pi^{(l')}_\theta, \boldsymbol{\sigma}^2_\theta, \tau^2_\theta)}. \tag{14}$$

As a final step in the model fitting procedure, we empirically compute (approximate) SNP-level posterior inclusion probabilities by marginalizing over the different grid combinations for $\boldsymbol{\pi}_\theta$. Namely,

$$\text{PIP}(j) \equiv \Pr[\gamma_{\theta j} = 1 \,|\, \mathbf{y}, \mathbf{X}] \approx \sum_{l=1}^{L}\lambda^{(l)}_\theta \Pr[\gamma_{\theta j} = 1 \,|\, \mathbf{y}, \mathbf{X}, \pi^{(l)}_\theta, \boldsymbol{\sigma}^2_\theta, \tau^2_\theta]. \tag{15}$$

This final step can be viewed as an analogy to Bayesian model averaging where marginal distributions are estimated via a weighted average of conditional distributions multiplied by importance weights [19,20,28].

2.2 Outer Layer (SNP-Set Level) Updates

In this section, we detail the posterior computation for parameters in the outer layer of the partially connected neural network. We are now interested in finding a distribution $q(\mathbf{w}, \boldsymbol{\gamma}_w)$ that approximates the true posterior $p(\mathbf{w}, \boldsymbol{\gamma}_w \,|\, \mathcal{D}, \boldsymbol{\theta})$. Here, it is important to note that, due to the integrative setup of the joint likelihood used in the BANNs framework, the true posterior for the weights in the outer layer is conditionally dependent upon the posterior estimates for the weights in the input layer. Since we assume that enriched SNP-sets contain at least one SNP with a nonzero effect, we consider a simpler family of variational distributions

$$q(w_g, \gamma_{wg}; \psi_g) = \begin{cases} \alpha_g \, \mathcal{N}(m_g, s_g^2) & \text{if} \quad \gamma_{wg} = 1 \\ (1-\alpha_g)\, \delta_0 & \text{if} \quad \gamma_{wg} = 0 \end{cases} \tag{16}$$

where $\psi_g = (\alpha_g, m_g, s_g^2)$ is used to describe a new set free parameters for the g-th SNP-set. Once again, our goal is to find the "best" factorized variational distribution amounts with free parameters $\boldsymbol{\psi}$ that minimize the Kullback-Leibler divergence between the exact and approximate posteriors. The specific class of variational distributions in Eq. (16) yields the following analytical expression for the lower bound on the outer layer (or SNP-set level)

$$\begin{aligned} \mathrm{LB}(\pi_w, \sigma_w^2, \tau_w^2 \,|\, \boldsymbol{\theta}) = & -\frac{N}{2}\log(2\pi\tau_w^2) - \frac{1}{2\tau_w^2}\|\mathbf{y} - \mathbf{H}(\boldsymbol{\theta})\boldsymbol{\beta}_w\|_2^2 - \frac{1}{2}\sum_{g=1}^{G}\{\mathbf{H}(\boldsymbol{\theta})^\intercal\mathbf{H}(\boldsymbol{\theta})\}_{gg}\,\mathbb{V}[w_g] \\ & - \sum_{g=1}^{G}\alpha_g\log\left(\frac{\alpha_g}{\pi_w}\right) - \sum_{g=1}^{G}(1-\alpha_g)\log\left(\frac{1-\alpha_g}{1-\pi_w}\right) \\ & + \frac{1}{2}\sum_{g=1}^{G}\alpha_g\left[1 + \log\left(\frac{s_g^2}{\sigma_w^2\tau_w^2}\right) - \frac{m_g^2 + s_g^2}{\sigma_w^2\tau_w^2}\right] \end{aligned} \tag{17}$$

where, similar to the input layer updates, $\tau_w^2 \approx \mathbb{V}[\mathbf{y} - \mathbf{H}(\boldsymbol{\theta})\boldsymbol{\beta}_w]$ estimates the variance of residual training error in the outer layer; the term $\boldsymbol{\beta}_w$ is a G-dimensional estimate of the posterior mean for $\mathbf{w}$ with elements $\beta_{wg} = \alpha_g m_g$ for the g-th SNP-set; the matrix $\mathbf{H}(\boldsymbol{\theta})^\intercal\mathbf{H}(\boldsymbol{\theta})$ is deterministically computed given posterior estimates of the weights $\boldsymbol{\theta}$ from the input layer, and $\mathbb{V}[w_g] = \alpha_g(m_g^2 + s_g^2) + \alpha_g^2 m_g^2$ is the variance of the g-th weight under the approximating distributional family in Eq. (16). We describe the explicit expectation and maximization steps of the approximate EM algorithm for the outer layer below.

1. **E-Step: Update the Variational Free Parameters.** In the E-step of the algorithm, we this time take the partial derivatives of the lower bound in Eq. (17) with respect to the free parameters in $\boldsymbol{\psi}$ and set them equal to zero. Solving for m_g, s_g^2, and α_g yields the following updates

$$m_g = \frac{s_g^2}{\tau_w^2}\left[(\mathbf{H}(\boldsymbol{\theta})^\intercal\mathbf{y})_g - \sum_{l\neq g}\{\mathbf{H}(\boldsymbol{\theta})^\intercal\mathbf{H}(\boldsymbol{\theta})\}_{lg}\,\beta_{wl}\right] \tag{18}$$

$$s_g^2 = \tau_w^2\left[\{\mathbf{H}(\boldsymbol{\theta})^\intercal\mathbf{H}(\boldsymbol{\theta})\}_{gg} + \frac{1}{\sigma_w^2}\right]^{-1} \tag{19}$$

$$\alpha_g = \mathrm{Sigmoid}\left(\log\left(\frac{\pi_w}{1-\pi_w}\right) + \log\left(\frac{s_g}{\sigma_w\tau_w}\right) + \frac{m_g^2}{2s_g^2}\right) \tag{20}$$

where $\alpha_g \approx \Pr[\gamma_{wg} = 1 \,|\, \mathbf{y}, \mathbf{X}, \boldsymbol{\theta}, \pi_w, \sigma_w^2, \tau_w^2]$ and, again, we set the sigmoid function to be the standard logistic function.

2. **M-Step: Update the Variance Hyper-Parameters.** In the M-step of the algorithm, we fix values of the variational free parameters and maximize the lower bound in Eq. (17) with respect to σ_w^2 and τ_w^2. Once again, this is done by solving for the roots σ_w^2 and τ_w^2 of the gradient which then yields the following updates

$$\sigma_w^2 = \left[\sum_{g=1}^{G} \alpha_g (m_g^2 + s_g^2)\right] \bigg/ \left(\tau_w^2 \sum_{g=1}^{G} \alpha_g\right) \tag{21}$$

$$\tau_w^2 = \left(N + \sum_{g=1}^{G} \alpha_g\right)^{-1} \left[\|\mathbf{y} - \mathbf{H}(\boldsymbol{\theta})\boldsymbol{\beta}_w\|_2^2 + \sum_{g=1}^{G} \{\mathbf{H}(\boldsymbol{\theta})^{\intercal}\mathbf{H}(\boldsymbol{\theta})\}_{gg} \mathbb{V}[w_g] + \frac{1}{\sigma_w^2} \sum_{g=1}^{G} \alpha_g (m_g^2 + s_g^2)\right] \tag{22}$$

where, again, N is equal to the dimensionality of the phenotypic response vector $\mathbf{y}$.

Similar to the algorithmic updates in the input layer, we account for our lack of *a priori* knowledge about the "correct" proportion of enriched SNP-sets by placing another exchangeable uniform hyper-prior distribution over an L-valued grid of possible values where $\{\pi_w^{(1)}, \ldots, \pi_w^{(L)}\} \in [1/G, 1]$. Here, we now use the variational lower bound in Eq. (17) to approximate the posterior distribution of π_w. As a final step in the model fitting procedure, we again conduct a Bayesian model averaging-like procedure by integrating over the different grid combinations for π_w and computing marginal posterior inclusion probabilities for each of the G-annotated SNP-sets as the following

$$\mathrm{PIP}(g) \equiv \Pr[\gamma_{wg} = 1 \,|\, \mathbf{y}, \mathbf{X}, \boldsymbol{\theta}] \approx \sum_{l=1}^{L} \lambda_w^{(l)} \Pr[\gamma_{wg} = 1 \,|\, \mathbf{y}, \mathbf{X}, \boldsymbol{\theta}, \pi_w^{(l)}, \sigma_w^2, \tau_w^2]. \tag{23}$$

where each importance weight $\lambda_w^{(l)}$ takes on a form similar to the normalized ratio described in Eq. (13).

3 Accounting for Non-Additive Genetic Effects

As mentioned in the main text, the BANNs framework models the proportion of phenotypic variance that is explained by sparse genetic effects (both additive and non-additive) [15]. This primarily done through the inclusion of the nonlinear Leaky ReLU activation function $h(\bullet)$ in the hidden layer [1]. In other areas of statistical genetics, similar nonlinear functions have been used to model non-additive effects that contribute to phenotypic variation [29–35]. For example, it has been shown that the Taylor series expansion of the Gaussian kernel function enumerates higher-order interaction terms between SNPs [36–39], thus alleviating potential combinatorial concerns with exhaustive searches [40]. The nonlinear ReLU function family shares this same property [41–43]. To see this, consider the general ReLU function for SNPs in the g-th SNP-set which is defined as

$$h(\mathbf{X}_g\boldsymbol{\theta}_g) = \max\left\{0, \sum_{j=1}^{|\mathcal{S}_g|} \mathbf{x}_j\theta_j + \mathbf{1}b_g^{(1)}\right\}. \tag{24}$$

Under this formulation, it becomes clear that the effect that any one SNP has in determining the output of the ReLU activation jointly depends on the effects of all other variants in the SNP-set — therefore, capturing the dependence or interaction between the inputs in the function. The SNP-set specific bias

terms $b_g^{(1)}$ allow each node in the hidden layer to change slope for different combinations of genotypes and provides more flexible estimation of broad-sense heritability. Theoretically, as more nodes and hidden layers are added to the network architecture, the model will have an even greater ability to account for non-additive genetic effects (acting similarly to classic Gaussian process regression methods) [44]. Through our simulation studies, we demonstrate the capability to accurately prioritize/rank associated SNPs and enriched SNP-sets in the BANNs framework, even in the presence of pairwise SNP-by-SNP interactions and population structure.

4 Estimating Phenotypic Variance Explained (PVE)

As described in the main text, we are able to provide an estimate of phenotypic variance explained (PVE) within the BANNs framework as the total proportion of phenotypic variance that is explained by sparse genetic effects (both additive and non-additive) [15]. Given the true values of the neural network parameters, we define this proportion on the SNP-level in the inner layer and SNP-set level in the outer layer as the following

$$\mathrm{PVE}(\boldsymbol{\theta}) \approx \frac{\mathbb{V}[\mathbf{X}\boldsymbol{\theta}]}{\mathbb{V}[\mathbf{y}]}, \qquad \mathrm{PVE}(\mathbf{w}) \approx \frac{\mathbb{V}[\mathbf{H}(\boldsymbol{\theta})\mathbf{w}]}{\mathbb{V}[\mathbf{y}]}, \tag{25}$$

where, as a reminder, $\mathbb{V}[\bullet]$ is the variance function and $\mathbf{H}(\boldsymbol{\theta}) = [h(\mathbf{X}_1\boldsymbol{\theta}_1 + \mathbf{1}b_1^{(1)}), \ldots, h(\mathbf{X}_G\boldsymbol{\theta}_G + \mathbf{1}b_G^{(1)})]$ denotes the matrix of deterministic nonlinear neurons in the hidden layer given estimates of the input layer weights. In practice, we estimate PVE using posterior values of the network parameters derived from the variational EM algorithm described in the previous section. Specifically, after averaging over the grid of different models, we use the (approximate) marginal posterior means $\boldsymbol{\beta}_\theta$ and $\boldsymbol{\beta}_w$ for the input and outer layer weights from Eqs. (8) and (16), respectively. We also approximate the variance of residual error that is observed during the training phase of both layers with estimates of τ_θ^2 and τ_w^2 from Eqs. (13) and (22). This yields the following empirical estimate for the PVE of complex traits

$$\mathrm{PVE}(\boldsymbol{\theta}) \approx \frac{\mathbb{V}[\mathbf{X}\boldsymbol{\beta}_\theta]}{\mathbb{V}[\mathbf{X}\boldsymbol{\beta}_\theta] + \tau_\theta^2}, \qquad \mathrm{PVE}(\mathbf{w}) \approx \frac{\mathbb{V}[\mathbf{H}(\boldsymbol{\beta}_\theta)\boldsymbol{\beta}_w]}{\mathbb{V}[\mathbf{H}(\boldsymbol{\beta}_\theta)\boldsymbol{\beta}_w] + \tau_w^2}, \tag{26}$$

where the matrix hidden neurons is empirically estimated as $\mathbf{H}(\boldsymbol{\beta}_\theta) = [h(\mathbf{X}_1\boldsymbol{\beta}_{\theta 1} + b_1^{(1)}), \ldots, h(\mathbf{X}_G\boldsymbol{\beta}_{\theta G} + b_G^{(1)})]$. Note that this formula is similar to the traditional form used for estimating PVE, except here we also consider the contribution of non-additive genetic effects through the nonlinear Leaky ReLU activation function $h(\bullet)$ [1]. Through various simulations, we demonstrate the ability to accurately estimate PVE in the BANNs framework under additive sparse architectures (see S26 and S27 Figs). We underestimate PVE in both polygenic traits and traits with pairwise SNP-by-SNP interactions, which we believe is caused by either (i) a misestimation of the approximate posterior mean for network weights or (ii) an over-estimation of the residual error variance during the variational EM algorithm. Similar observations have been noted when using variational inference [9,19,25,45]. Results from other work also suggest that the sparsity assumption on the SNP-level effects can lead to the underestimation of the PVE [14,15].

5 Ablation Test and Analysis

To investigate how choices in the model setup contribute to variable selection, we performed an "ablation analysis" where we modified parts of the BANNs framework independently and observed their direct effect on model performance. We considered two different modifications to our model: (1) removing the activation function and training a fully linear hierarchical model, and (2) removing the approximate Bayesian model averaging approach and updating the probabilities π_θ and π_w as additional parameters in

the variational EM algorithm. In the normal BANNs setup, we initialize L different models with varying priors for inclusion probabilities specified over a grid $\{\pi_\theta^{(1)}, \ldots, \pi_\theta^{(L)}\} \in [1/J, 1]$ and $\{\pi_w^{(1)}, \ldots, \pi_w^{(L)}\} \in [1/G, 1]$, respectively. However, in the case of the latter ablation modification, we initialize $\pi_\theta = 1/J$ and $\pi_w = 1/G$ as an analogy to the "single causal variant" assumption frequently used in fine mapping [46]. Next, we update their values in the M-step of the algorithm according to the following analytic expressions

$$\frac{\pi_\theta}{1-\pi_\theta} = \frac{\sum_j \sum_k \alpha_{jk}}{\sum_j \sum_k (1-\alpha_{jk})}, \qquad \frac{\pi_w}{1-\pi_w} = \frac{\sum_g \alpha_g}{\sum_g (1-\alpha_g)}. \tag{27}$$

Results presented in the main text and Supporting Information (see S25 Fig) are shown using simulations with the self-identified "white British" ancestry cohort from the UK Biobank on synthetic traits that have broad-sense heritability $H^2 = 0.6$. Each plot combines results from 100 simulated replicates (see Section 9 for details on the setup for the simulation study).

6 Data Quality Control Procedures for Stock of Mice

Some of the real data analysis results in this work made use of GWA data from the Wellcome Trust Centre for Human Genetics (`http://mtweb.cs.ucl.ac.uk/mus/www/mouse/index.shtml`). This study contains N = 1,814 heterogenous stock of mice from 85 families (all descending from eight inbred progenitor strains) [47], and 131 quantitative traits that are classified into 6 broad categories including behavior, diabetes, asthma, immunology, haematology, and biochemistry (`http://mtweb.cs.ucl.ac.uk/mus/www/GSCAN/index.shtml/index.old.shtml`). In the main text, we focused on six specific phenotypes from these categories including: body mass index (BMI) (`Obesity.BMI`), body weight (`Glucose.BodyWeight`), percentage of CD8+ cells (`Imm.PctCD8`), mean corpuscular hemoglobin (MCH) (`Haem.MCH`), high-density lipoprotein content (`Biochem.HDL`), and low-density lipoprotein content (`Biochem.LDL`). All phenotypes were previously corrected for sex, age, body weight, season, year, and cage effects [47]. For individuals with missing genotypes, we imputed values by the mean genotype of that SNP in their corresponding family. Only polymorphic SNPs with minor allele frequency above 5% were kept for the analyses. This left a total of J = 10,227 autosomal SNPs that were available for all mice. For annotations, we used the Mouse Genome Informatics database (`http://www.informatics.jax.org`) to map SNPs to the closest neighboring gene(s). Here, pseudogenes, quantitative trait loci (QTL), and genes with only 1 annotated SNP within their boundary were excluded from the analyses. Unannotated SNPs located within the same genomic region were labeled as being within the "intergenic region" between two genes. Altogether, a total of G = 1,925 SNP-sets were analyzed.

7 Data Quality Control Procedures for Framingham Heart Study

The other real data analysis results made use of human GWA data from the Framingham Heart Study (`https://www.ncbi.nlm.nih.gov/gap`) [48]. This study originally contains N = 6,950 individuals and J = 394,174 SNPs. For quality control on these data, we removed (i) SNPs with minor allele frequency less than 2.5%, (ii) SNPs not in Hardy-Weinberg Equilibrium (Fisher's exact test $P > 1 \times 10^{-4}$), and (iii) proximal SNPs in high linkage disequilibrium (using the flag `--indep-pairwise 50 5 0.8` with PLINK 1.9 [49]). This resulted in a final dataset containing J = 372,131 SNPs, where any missing values for a given SNP were imputed by using the estimated mean genotype of that SNP. Next, we used the NCBI's Reference Sequence (RefSeq) database in the UCSC Genome Browser [50] to annotate SNPs with appropriate genes. Recall that in the real data analysis, we define genes with boundaries in two ways: (a) we use the UCSC gene boundary definitions directly, or (b) we augment the gene boundaries by adding SNPs within a ±500 kilobase (kb) buffer to account for possible regulatory elements. Genes with only 1 SNP within their boundary were excluded from either analysis. Unannotated SNPs located

within the same genomic region were labeled as being within the "intergenic region" between two genes. Altogether, a total of $G = 18{,}364$ SNP-sets were analyzed—which included 8,658 intergenic SNP-sets and 9,706 annotated genes—using the UCSC boundaries. When including the 500kb buffer, a total of $G = 35{,}871$ SNP-sets were analyzed.

8 Data Quality Control Procedures for UK Biobank

The simulation results and additional lipoprotein study presented in the main text made use of imputed data released from the UK Biobank [51]. Quality control procedures for these data are as follows. First, we only studied individuals who self-identified as being of European ancestry. From this cohort, we further excluded individuals identified by the UK Biobank to have high heterozygosity, excessive relatedness, or aneuploidy (1,550 individuals removed). We also removed individuals whose kinship coefficient was greater than 0.0442 (i.e., close relatives). Next, we removed (i) monomorphic SNPs, (ii) SNPs with minor allele frequency less than 2.5%, (iii) SNPs not in Hardy-Weinberg Equilibrium (Fisher's exact test $P > 1 \times 10^{-6}$), (iv) SNPs with missingness greater than 1%, and (v) SNPs in high linkage disequilibrium (using the flag `--indep-pairwise 50 5 0.9` with PLINK 1.9 [49]). After all QC steps, we had a final dataset of $N = 349{,}414$ individuals and $J = 1{,}070{,}306$ SNPs. Next, we used the NCBI's Reference Sequence (RefSeq) database in the UCSC Genome Browser [50] to annotate SNPs with appropriate genes. Again, in the real data analysis, we define genes with boundaries in two ways: (a) we use the UCSC gene boundary definitions directly, or (b) we augment the gene boundaries by adding SNPs within a ± 500 kilobase (kb) buffer to account for possible regulatory elements. Genes with only 1 SNP within their boundary were excluded from either analysis. Unannotated SNPs located within the same genomic region were labeled as being within the "intergenic region" between two genes. Altogether, a total of $G = 28{,}644$ SNP-sets were kept for analysis using the UCSC boundaries and a total of $G = 35{,}849$ SNP-sets were kept for analysis when including the 500kb buffer.

9 Simulation Setup and Scenarios

In our simulation studies, we used the following general simulation scheme to generate quantitative traits using real genotype data on chromosome 1 from ten thousand randomly sampled individuals of European ancestry in the UK Biobank [51]. We consider two different data compositions. In the first, we simulate synthetic traits only using individuals who self-identify as being of "white British" ancestry. In the second, we simulate phenotypes by random subsampling 3,000 individuals who self-identify as being of "white British" ancestry, 3,000 individuals who self-identify as being of "white Irish" ancestry, and 4,000 individuals who identify as being of "any other white background". Note that the latter composition introduces additional, yet cryptic, population structure into the problem. In the main text and Supporting information (i.e., Figs 2, 3, and S2-S27, and S1-S8 Tables), we refer to these datasets as the "British" and "European" cohorts, respectively.

The setup to generate synthetic traits follows mostly from previous studies [13,38–40]. We will denote this genotype matrix as $\mathbf{X}$, with $\mathbf{x}_j$ denoting the genotypic vector for the j-th SNP. Following quality control procedures detailed in the previous section, our simulations included $J = 36{,}518$ SNPs distributed across genome. Again, we used the NCBI's RefSeq database in the UCSC Genome Browser to assign SNPs to genes which resulted in 1,408 genes to be used in the simulation study. We also consider the unnanotated SNPs between two genes to be located within intergenic regions. Altogether, a total of $G = 2{,}816$ SNP-sets were analyzed.

After the annotation step, we assume that all simulated traits have been standardized such that $\mathbb{V}[\mathbf{y}] = 1$ and that all observed genetic effects explain a fixed proportion of this value (i.e., broad-sense heritability, H^2). To be explicit, one can equate the total PVE in these simulations to H^2. Next, we use

the $N \times J$ matrix of genotypes $\mathbf{X}$ to generate real-valued phenotypes that mirror genetic architectures affected by a combination of linear (additive) and interaction (epistatic) effects. We randomly select a certain percentage of truly associated SNP-sets and denote the SNPs that they contain as $\mathcal{C}$. Within $\mathcal{C}$, we select causal SNPs in a way such that each associated SNP-set contains at least two SNPs with non-zero effects. The additive effect size for all causal SNPs are assumed to come from a standard normal distribution, $\boldsymbol{\theta} \sim \mathcal{N}(\mathbf{0}, \mathbf{I})$. Next, we create a separate matrix $\mathbf{W}$ which holds the pairwise interactions between the causal SNPs in enriched SNP-sets. This is done by taking the Hadamard (element-wise) product between genotypic vectors of SNPs within $\mathcal{C}$. The corresponding interaction effect sizes are drawn as $\boldsymbol{\varphi} \sim \mathcal{N}(\mathbf{0}, \mathbf{I})$. We scale both the additive and pairwise genetic effects so that collectively they explain a fixed proportion of genetic variance. Namely, the additive effects make up $\rho\%$ while the pairwise interactions make up the remaining $(1-\rho)\%$. Alternatively, the proportion of the heritability explained by additivity is said to be $\mathbb{V}[\sum \mathbf{x}_c\theta_c] = \rho H^2$, while the proportion detailed by genetic interactions is given as $\mathbb{V}[\mathbf{W}\boldsymbol{\varphi}] = (1-\rho)H^2$. We consider two choices for the parameter $\rho = \{0.5, 1\}$. Intuitively, $\rho = 1$ represents the limiting case where the variation of a trait is driven by solely additive effects. For $\rho = 0.5$, the additive and pairwise interaction effects are assumed to equally contribute to the phenotypic variance. Once we obtain the final effect sizes for all causal variants, we draw normally distributed random errors as $\boldsymbol{\varepsilon} \sim \mathcal{N}(\mathbf{0}, \mathbf{I})$ to make up the remaining percentage of the total variance. Quantitative continuous traits are then generated under the following general linear model:

$$\mathbf{y} = \sum_{c \in \mathcal{C}} \mathbf{x}_c\theta_c + \mathbf{W}\boldsymbol{\varphi} + \boldsymbol{\varepsilon}. \tag{28}$$

Given the simulation procedure above, we randomly sample $N = 10{,}000$ individuals and simulate a wide range of scenarios for comparing the performance of both SNP and SNP-set level association methods. Here, we vary the following simulation parameters:

- Broad-sense heritability: $H^2 = 0.2$ and 0.6;
- Contribution of interaction effects: $(1-\rho) = 0$ and 0.5;
- Percentage of associated SNP-sets: 1% (sparse architecture) and 10% (polygenic architecture);

Lastly, we set the number of causal SNPs with non-zero effects to be some fixed percentage of all SNPs located within the selected associated SNP-sets. We set this percentage to be 1% in the 1% associated SNP-set case, and 10% in the 10% associated SNP-set case. All performance comparisons are based on 100 different simulated runs for each parameter combination. For evaluating the performance of each method, we assessed the following:

- The power and false discovery rates when identifying causal SNPs or associated SNP-sets at a Bonferroni-corrected threshold for frequentist approaches ($P = 0.05/36518 = 1.37 \times 10^{-6}$ at the SNP-level and $P = 0.05/2816 = 1.78 \times 10^{-5}$ at the SNP-set level) or according to the median probability model for Bayesian methods (posterior enrichment probability > 0.5) [52];
- The ability to rank true positive (TP) genes over false positives (FP) via receiver operating characteristic (ROC) and precision-recall curves.

All figures and tables show the mean performances (and standard errors) across all simulated replicates.

10 Software Details

Source code for the BANNs framework is freely available at `https://github.com/lcrawlab/BANNs` and is licensed under the GNU General Public License (version 3.0). We have released two versions of the

BANNs software: one implemented within `Python 3` (release version 3.7.7) and other within `R` (compatible with versions 3.3.2 through 3.6.3). The BANNs GitHub repository includes example data, documentation, and instructions for how to execute the code within both coding languages. Results in the main text and Supporting Information are based on the `Python 3` implementation which depends on the `pandas` library (version 1.0.1) [53] for automatically creating partial neural network architectures based on the biological annotations provided by the user; the `NumPy` (version 1.18-19) [54] and `Numba` (version 0.48.0) [55] packages for efficient matrix operations; and the `multiprocessing` library (version 2.6) [56] for parallelizing posterior computation over multiple threads and providing faster execution. Training, estimation of the network parameters, and optimization was done by using an Adam optimizer [57] in `TensorFlow` (version 1.5). While the software can be run directly using the source code, it can also be installed as a package through `pip` with the command: `pip3 install BANNs`. All dependencies are also automatically installed with the package.

The `R` implementation uses the `dplyr` package (version 0.8.5) [58] for automatically creating partial neural network architectures based on the biological annotations provided by the user; the `Matrix` package (version 1.2-18) [59] for efficient matrix operations; and the `doParallel` (version 1.0.15) [60], `forEach` (version 1.4.8) [61], `iterators` (version 1.0.12) [62], and standard `parallel` packages for parallelized execution of the variational expectation-maximization algorithm. Similarly, the `R` implementation of the software can be run by directly downloading the source code or it can be installed using `devtools` [63] with the commands: `devtools::install("lcrawlab/BANNs")` and `library(BANNs)`.

Software Details for Competing Approaches. In this work, comparisons to SNP-level association mapping methods were made using software for CAVIAR (version 2.0.0; `http://genetics.cs.ucla.edu/caviar/`), FINEMAP (version 1.4; `http://www.christianbenner.com`), and SuSiE (version 0.9.0; `https://github.com/stephenslab/susieR`). Comparisons to SNP-set mapping methods were made using software for GBJ (version 0.5.3; `https://cran.r-project.org/web/packages/GBJ/`), GSEA (`https://www.nr.no/en/projects/software-genomics`), MAGMA (version 1.07b; `https://ctg.cncr.nl/software/magma`), PEGASUS (version 1.3.0; `https://github.com/ramachandran-lab/PEGASUS`), RSS (version 1.0.0; `https://github.com/stephenslab/rss`), and SKAT (version 1.3.2.1; `https://www.hsph.harvard.edu/skat`), which are also publicly available. All software for competing methods were fit using the default settings, unless otherwise stated in the main text and Supporting Information.

11 Pseudocode for Biologically Annotated Neural Networks

Algorithm 1 BANNs Model with Individual Level Data

Input genotype data $\mathbf{X}$, continuous trait $\mathbf{y}$, and annotations $\{\mathcal{S}_1, \ldots, \mathcal{S}_G\}$.
Choose the number of models L, number of maximum iterations T, and tolerance parameter ϵ.
Set up the L-grid of possible values $\{\pi_\theta^{(1)}, \ldots, \pi_\theta^{(L)}\} \in [1/J, 1]$ and $\{\pi_w^{(1)}, \ldots, \pi_w^{(L)}\} \in [1/G, 1]$ for the inner and outer layer, respectively.
Randomly initialize variational parameters $\{\alpha_{jk}, m_{jk}, s_{jk}^2\}_{k=1}^K$, $\{\sigma_{\theta k}^2\}_{k=1}^K$, and τ_θ^2 for the inner layer.
Randomly initialize variational parameters $\{\alpha_g, m_g, s_g^2\}$, σ_w^2, and τ_w^2 for the outer layer.
for each $\pi_\theta^{(l)} \in \{\pi_\theta^{(1)}, \ldots, \pi_\theta^{(L)}\}$ and $\pi_w^{(l)} \in \{\pi_w^{(1)}, \ldots, \pi_w^{(L)}\}$ **do**
    Compute inner lower bound `LB_inner_new`.
  for $t = 1 \to T$ **do** ▷ Inner Layer Updates
      Set `LB_inner` = `LB_inner_new`.
      Update variational parameters $\{\alpha_{jk}, m_{jk}, s_{jk}^2\}_{k=1}^K$ for $j = 1, \ldots, J$ SNPs. ▷ E-Step
      Update hyper-parameters parameters $\{\sigma_{\theta k}^2\}_{k=1}^K$ and τ_θ^2. ▷ M-Step
      Update lower bound `LB_inner_new`.
    if `LB_inner_new` − `LB_inner` $\leq \epsilon$ **then**
        Save `LB_inner` = `LB_inner_new`.
        Break
    end if
  end for
    Compute hidden layer neurons $\mathbf{H}(\boldsymbol{\theta})$.
    Compute outer lower bound `LB_outer_new`.
  for $t = 1 \to T$ **do** ▷ Outer Layer Updates
      Set `LB_outer` = `LB_outer_new`.
      Update variational parameters $\{\alpha_g, m_g, s_g^2\}$ for $g = 1, \ldots, G$ SNP-sets. ▷ E-Step
      Update hyper-parameters parameters σ_w^2 and τ_w^2. ▷ M-Step
      Update lower bound `LB_outer_new`.
    if `LB_outer_new` − `LB_outer` $\leq \epsilon$ **then**
        Save `LB_outer` = `LB_inner_outer`.
        Break
    end if
  end for
end for each
Compute normalized importance weights $\lambda_\theta^{(l)}$ and $\lambda_g^{(l)}$ for $l = 1, \ldots, L$ models.
Compute (marginal) posterior means $\boldsymbol{\beta}_\theta$ and $\boldsymbol{\beta}_w$ for network weights $\boldsymbol{\theta}$ and $\mathbf{w}$, respectively.
Compute (marginal) posterior inclusion probabilities $\text{PIP}(\boldsymbol{\theta})$ and $\text{PIP}(\mathbf{w})$.
Compute the phenotypic variance explained by the input and hidden layers $\text{PVE}(\boldsymbol{\theta})$ and $\text{PVE}(\mathbf{w})$.
Return $\{\boldsymbol{\beta}_\theta, \boldsymbol{\beta}_w, \text{PIP}(\boldsymbol{\theta}), \text{PIP}(\mathbf{w}), \text{PVE}(\boldsymbol{\theta}), \text{PVE}(\mathbf{w})\}$.

Algorithm 2 BANN-SS Model with GWA Summary Statistics

Input LD matrix $\mathbf{R}$, OLS effect size estimates $\widehat{\boldsymbol{\theta}}$, and annotations $\{\mathcal{S}_1, \ldots, \mathcal{S}_G\}$.
Choose the number of models L, number of maximum iterations T, and tolerance parameter ϵ.
Set up the L-grid of possible values $\{\pi_\theta^{(1)}, \ldots, \pi_\theta^{(L)}\} \in [1/J, 1]$ and $\{\pi_w^{(1)}, \ldots, \pi_w^{(L)}\} \in [1/G, 1]$ for the inner and outer layer, respectively.
Randomly initialize variational parameters $\{\alpha_{jk}, m_{jk}, s_{jk}^2\}_{k=1}^K$, $\{\sigma_{\theta k}^2\}_{k=1}^K$, and τ_θ^2 for the inner layer.
Randomly initialize variational parameters $\{\alpha_g, m_g, s_g^2\}$, σ_w^2, and τ_w^2 for the outer layer.
for each $\pi_\theta^{(l)} \in \{\pi_\theta^{(1)}, \ldots, \pi_\theta^{(L)}\}$ and $\pi_w^{(l)} \in \{\pi_w^{(1)}, \ldots, \pi_w^{(L)}\}$ **do**
        Compute inner lower bound `LB_inner_new`.
    for $t = 1 \to T$ **do** ▷ Inner Layer Updates
            Set `LB_inner` = `LB_inner_new`.
            Update variational parameters $\{\alpha_{jk}, m_{jk}, s_{jk}^2\}_{k=1}^K$ for $j = 1, \ldots, J$ SNPs. ▷ E-Step
            Update hyper-parameters parameters $\{\sigma_{\theta k}^2\}_{k=1}^K$ and τ_θ^2. ▷ M-Step
            Update lower bound `LB_inner_new`.
        if `LB_inner_new` − `LB_inner` $\leq \epsilon$ **then**
                Save `LB_inner` = `LB_inner_new`.
                Break
        end if
    end for
        Compute hidden layer neurons $\mathbf{H}(\boldsymbol{\theta})$.
        Compute outer lower bound `LB_outer_new`.
    for $t = 1 \to T$ **do** ▷ Outer Layer Updates
            Set `LB_outer` = `LB_outer_new`.
            Update variational parameters $\{\alpha_g, m_g, s_g^2\}$ for $g = 1, \ldots, G$ SNP-sets. ▷ E-Step
            Update hyper-parameters parameters σ_w^2 and τ_w^2. ▷ M-Step
            Update lower bound `LB_outer_new`.
        if `LB_outer_new` − `LB_outer` $\leq \epsilon$ **then**
                Save `LB_outer` = `LB_inner_outer`.
                Break
        end if
    end for
end for each
Compute normalized importance weights $\lambda_\theta^{(l)}$ and $\lambda_g^{(l)}$ for $l = 1, \ldots, L$ models.
Compute (marginal) posterior means $\boldsymbol{\beta}_\theta$ and $\boldsymbol{\beta}_w$ for network weights $\boldsymbol{\theta}$ and $\mathbf{w}$, respectively.
Compute (marginal) posterior inclusion probabilities $\mathrm{PIP}(\boldsymbol{\theta})$ and $\mathrm{PIP}(\mathbf{w})$.
Compute the phenotypic variance explained by the input and hidden layers $\mathrm{PVE}(\boldsymbol{\theta})$ and $\mathrm{PVE}(\mathbf{w})$.
Return $\{\boldsymbol{\beta}_\theta, \boldsymbol{\beta}_w, \mathrm{PIP}(\boldsymbol{\theta}), \mathrm{PIP}(\mathbf{w}), \mathrm{PVE}(\boldsymbol{\theta}), \mathrm{PVE}(\mathbf{w})\}$.

References

1. Xu B, Wang N, Chen T, Li M. Empirical evaluation of rectified activations in convolutional network; 2015. ArXiv.

2. Wang L, Zhang B, Wolfinger RD, Chen X. An integrated approach for the analysis of biological pathways using mixed models. PLoS Genet. 2008;4(7):e1000115. Available from: https://doi.org/10.1371/journal.pgen.1000115.

3. Califano A, Butte AJ, Friend S, Ideker T, Schadt E. Leveraging models of cell regulation and GWAS data in integrative network-based association studies. Nat Genet. 2012;44(8):841–847. Available from: https://doi.org/10.1038/ng.2355.

4. Carbonetto P, Stephens M. Integrated enrichment analysis of variants and pathways in genome-wide association studies indicates central role for IL-2 signaling genes in type 1 diabetes, and cytokine signaling genes in Crohn's disease. PLoS Genet. 2013;9(10):e1003770. Available from: https://doi.org/10.1371/journal.pgen.1003770.

5. Yang J, Fritsche LG, Zhou X, Abecasis G, Consortium IARMDG. A scalable Bayesian method for integrating functional information in genome-wide association studies. Am J Hum Genet. 2017;101(3):404–416.

6. van der Wijst MGP, de Vries DH, Brugge H, Westra HJ, Franke L. An integrative approach for building personalized gene regulatory networks for precision medicine. Genome Med. 2018;10(1):96. Available from: https://doi.org/10.1186/s13073-018-0608-4.

7. Zhu X, Stephens M. Large-scale genome-wide enrichment analyses identify new trait-associated genes and pathways across 31 human phenotypes. Nat Comm. 2018;9(1):4361.

8. Kichaev G, Bhatia G, Loh PR, Gazal S, Burch K, Freund MK, et al. Leveraging Polygenic Functional Enrichment to Improve GWAS Power. Am J Hum Genet. 2019;104(1):65–75. Available from: https://pubmed.ncbi.nlm.nih.gov/30595370.

9. Zeng P, Zhou X. Non-parametric genetic prediction of complex traits with latent Dirichlet process regression models. Nat Comm. 2017;8:456. Available from: https://doi.org/10.1038/s41467-017-00470-2.

10. Moser G, Lee SH, Hayes BJ, Goddard ME, Wray NR, Visscher PM. Simultaneous discovery, estimation and prediction analysis of complex traits using a Bayesian mixture model. PLoS Genet. 2015;11(4):e1004969. Available from: https://pubmed.ncbi.nlm.nih.gov/25849665.

11. Zhang Y, Qi G, Park JH, Chatterjee N. Estimation of complex effect-size distributions using summary-level statistics from genome-wide association studies across 32 complex traits. Nat Genet. 2018;50(9):1318–1326.

12. Lloyd-Jones LR, Zeng J, Sidorenko J, Yengo L, Moser G, Kemper KE, et al. Improved polygenic prediction by Bayesian multiple regression on summary statistics. Nat Comm. 2019;10(1):5086. Available from: https://doi.org/10.1038/s41467-019-12653-0.

13. Cheng W, Ramachandran S, Crawford L. Estimation of non-null SNP effect size distributions enables the detection of enriched genes underlying complex traits. PLoS Genet. 2020;16(6):e1008855. Available from: https://doi.org/10.1371/journal.pgen.1008855.

14. Guan Y, Stephens M. Bayesian variable selection regression for genome-wide association studies and other large-scale problems. Ann Appl Stat. 2011;5(3):1780–1815. Available from: https://projecteuclid.org:443/euclid.aoas/1318514285.

15. Zhou X, Carbonetto P, Stephens M. Polygenic modeling with Bayesian sparse linear mixed models. PLoS Genet. 2013;9(2):e1003264.

16. Zhu X, Stephens M. Bayesian large-scale multiple regression with summary statistics from genome-wide association studies. Ann Appl Stat. 2017;11(3):1561–1592. Available from: https://projecteuclid.org:443/euclid.aoas/1507168840.

17. George EI, McCulloch RE. Variable selection via Gibbs sampling. J Am Stat Assoc. 1993;88(423):881–889.

18. Blei DM, Jordan MI. Variational inference for Dirichlet process mixtures. Bayesian Anal. 2006;1(1):121–143.

19. Carbonetto P, Stephens M. Scalable variational inference for Bayesian variable selection in regression, and its accuracy in genetic association studies. Bayesian Anal. 2012;7(1):73–108.

20. Carbonetto P, Zhou X, Stephens M. varbvs: Fast variable selection for large-scale regression; 2017. ArXiv.

21. Jordan MI, Ghahramani Z, Jaakkola TS, Saul LK. An introduction to variational methods for graphical models. Mach Learn. 1999;37(2):183–233.

22. Bishop CM. Pattern recognition and machine learning. Springer; 2006.

23. Ormerod JT, Wand MP. Explaining variational approximations. Am Stat. 2010;64(2):140–153.

24. Grimmer J. An introduction to Bayesian inference via variational approximations. Political Anal. 2011;19(1):32–47.

25. Blei DM, Kucukelbir A, McAuliffe JD. Variational inference: A review for statisticians. J Am Stat Assoc. 2017;112(518):859–877.

26. Wand MP, Ormerod JT, Padoan SA, Frühwirth R. Mean field variational Bayes for elaborate distributions. Bayesian Anal. 2011;6(4):847–900.

27. Pham TH, Ormerod JT, Wand MP. Mean field variational Bayesian inference for nonparametric regression with measurement error. Comput Stat Data Anal. 2013;68:375–387.

28. Hoeting JA, Madigan D, Raftery AE, Volinsky CT. Bayesian model averaging: a tutorial (with comments by M. Clyde, David Draper and E. I. George, and a rejoinder by the authors. Statist Sci. 1999;14(4):382–417. Available from: https://projecteuclid.org:443/euclid.ss/1009212519.

29. de los Campos G, Naya H, Gianola D, Crossa J, Legarra A, Manfredi E, et al. Predicting quantitative traits with regression models for dense molecular markers and pedigree. Genetics. 2009;182(1):375–385. Available from: http://www.genetics.org/content/182/1/375.abstract.

30. de los Campos G, Gianola D, Rosa GJM, Weigel KA, Crossa J. Semi-parametric genomic-enabled prediction of genetic values using reproducing kernel Hilbert spaces methods. Genet Res (Camb). 2010;92(4):295–308.

31. Morota G, Gianola D. Kernel-based whole-genome prediction of complex traits: a review. Front Genet. 2014;5:363.

32. Swain PS, Stevenson K, Leary A, Montano-Gutierrez LF, Clark IBN, Vogel J, et al. Inferring time derivatives including cell growth rates using Gaussian processes. Nat Comm. 2016;7(1):13766. Available from: https://doi.org/10.1038/ncomms13766.

33. Weissbrod O, Geiger D, Rosset S. Multikernel linear mixed models for complex phenotype prediction. Genome Res. 2016;26(7):969–979. Available from: http://genome.cshlp.org/content/26/7/969.abstract.

34. Cheng L, Ramchandran S, Vatanen T, Lietzén N, Lahesmaa R, Vehtari A, et al. An additive Gaussian process regression model for interpretable non-parametric analysis of longitudinal data. Nat Comm. 2019;10(1):1–11.

35. Weissbrod O, Kaufman S, Golan D, Rosset S. Maximum likelihood for Gaussian process classification and generalized linear mixed models under case-control sampling. J Mach Learn Res. 2019;20(108):1–30.

36. Cotter A, Keshet J, Srebro N. Explicit approximations of the Gaussian kernel; 2011. ArXiv.

37. Jiang Y, Reif JC. Modeling epistasis in genomic selection. Genetics. 2015;201:759–768.

38. Crawford L, Wood KC, Zhou X, Mukherjee S. Bayesian approximate kernel regression with variable selection. J Am Stat Assoc. 2018;113(524):1710–1721.

39. Crawford L, Flaxman SR, Runcie DE, West M. Variable prioritization in nonlinear black box methods: A genetic association case study. Ann Appl Stat. 2019;13(2):958–989.

40. Crawford L, Zeng P, Mukherjee S, Zhou X. Detecting epistasis with the marginal epistasis test in genetic mapping studies of quantitative traits. PLoS Genet. 2017;13(7):e1006869. Available from: https://doi.org/10.1371/journal.pgen.1006869.

41. Tsang M, Cheng D, Liu Y. Detecting statistical interactions from neural network weights. In: International Conference on Learning Representations; 2018. p. 1–21.

42. Tsang M, Liu H, Purushotham S, Murali P, Liu Y. Neural interaction transparency (nit): Disentangling learned interactions for improved interpretability. In: Advances in Neural Information Processing Systems; 2018. p. 5804–5813.

43. Murdoch WJ, Singh C, Kumbier K, Abbasi-Asl R, Yu B. Definitions, methods, and applications in interpretable machine learning. Proc Natl Acad Sci USA. 2019;116(44):22071. Available from: http://www.pnas.org/content/116/44/22071.abstract.

44. Lee J, Sohl-dickstein J, Pennington J, Novak R, Schoenholz S, Bahri Y. Deep Neural Networks as Gaussian Processes. In: International Conference on Learning Representations; 2018. Available from: https://openreview.net/forum?id=B1EA-M-0Z.

45. Giordano R, Broderick T, Jordan MI. Covariances, robustness and variational bayes. J Mach Learn Res. 2018;19(1):1981–2029.

46. Wang G, Sarkar A, Carbonetto P, Stephens M. A simple new approach to variable selection in regression, with application to genetic fine-mapping. J R Stat Soc B. 2020;82:1273–1300. BioRxiv. Available from: http://biorxiv.org/content/early/2019/07/29/501114.abstract.

47. Valdar W, Solberg LC, Gauguier D, Burnett S, Klenerman P, Cookson WO, et al. Genome-wide genetic association of complex traits in heterogeneous stock mice. Nat Genet. 2006;38(8):879–887. Available from: http://dx.doi.org/10.1038/ng1840.

48. Splansky GL, Corey D, Yang Q, Atwood LD, Cupples LA, Benjamin EJ, et al. The Third Generation Cohort of the National Heart, Lung, and Blood Institute's Framingham Heart Study: design, recruitment, and initial examination. Am J Epidemiol. 2007;165(11):1328–1335.

49. Purcell S, Neale B, Todd-Brown K, Thomas L, Ferreira MA, Bender D, et al. PLINK: a tool set for whole-genome association and population-based linkage analyses. Am J Hum Genet. 2007;81(3):559–575.

50. Pruitt KD, Tatusova T, Maglott DR. NCBI Reference Sequence (RefSeq): a curated non-redundant sequence database of genomes, transcripts and proteins. Nucleic Acids Res. 2005;33(Database issue):D501–4.

51. Bycroft C, Freeman C, Petkova D, Band G, Elliott LT, Sharp K, et al. The UK Biobank resource with deep phenotyping and genomic data. Nature. 2018;562(7726):203–209. Available from: https://doi.org/10.1038/s41586-018-0579-z.

52. Barbieri MM, Berger JO. Optimal predictive model selection. Ann Statist. 2004;32(3):870–897. Available from: http://projecteuclid.org/euclid.aos/1085408489.

53. McKinney W. Data Structures for Statistical Computing in Python. In: van der Walt S, Millman J, editors. Proceedings of the 9th Python in Science Conference; 2010. p. 51–56.

54. Walt Svd, Colbert SC, Varoquaux G. The NumPy array: a structure for efficient numerical computation. Comput Sci Eng. 2011;13(2):22–30. Available from: https://aip.scitation.org/doi/abs/10.1109/MCSE.2011.37.

55. Lam SK, Pitrou A, Seibert S. Numba: A LLVM-Based Python JIT Compiler. In: Proceedings of the Second Workshop on the LLVM Compiler Infrastructure in HPC. LLVM '15. New York, NY, USA: Association for Computing Machinery; 2015. p. 1–6. Available from: https://doi.org/10.1145/2833157.2833162.

56. Mckerns MM, Sullivan T, Fang A, Aivazis MA. Aivazis, Building a framework for predictive science. In: Proceedings of the 10th Python in Science Conference. Citeseer; 2011. .

57. Kingma DP, Ba J. Adam: a method for stochastic optimization; 2014.

58. Wickham H, François R, Henry L, Müller K. dplyr: A Grammar of Data Manipulation; 2018. R package version 0.8.5. Available from: https://CRAN.R-project.org/package=dplyr.

59. Bates D, Maechler M. Matrix: Sparse and Dense Matrix Classes and Methods; 2019. R package version 1.2-18. Available from: https://CRAN.R-project.org/package=Matrix.

60. Corporation M, Weston S. doParallel: Foreach Parallel Adaptor for the 'parallel' Package; 2019. R package version 1.0.15. Available from: https://CRAN.R-project.org/package=doParallel.

61. Microsoft, Weston S. foreach: Provides Foreach Looping Construct; 2020. R package version 1.4.8. Available from: https://CRAN.R-project.org/package=foreach.

62. Analytics R, Weston S. iterators: Provides Iterator Construct; 2019. R package version 1.0.12. Available from: https://CRAN.R-project.org/package=iterators.

63. Wickham H, Hester J, Chang W. devtools: Tools to Make Developing R Packages Easier; 2019. R package version 2.2.1. Available from: https://CRAN.R-project.org/package=devtools.
